# Enzyme promiscuity-driven co-production of flavonoid 7-*O*-glycosides in engineered *Saccharomyces cerevisiae*

**DOI:** 10.1016/j.synbio.2026.01.033

**Published:** 2026-02-12

**Authors:** Xinjia Tan, Shasha Zuo, Fanglin Hu, Zhiqiang Xiao, Yongtong Wang, Siqi Zhang, Qiyuan Lu, Yifei Zhao, Jiaxu Chen, Liusha Fan, Juan Liu, Yang Shan

**Affiliations:** a Hunan Institute of Agricultural Products Processing and Quality Safety, Dongting Laboratory, Hunan Academy of Agricultural Sciences, Changsha, 410125, China; b Yuelushan Laboratory, Changsha, 410128, China; c Longping Agricultural College, Hunan University, Changsha, 410125, China; d College of Food Science and Nutritional Engineering, China Agricultural University, Beijing, 100083, China

**Keywords:** 7-*O*-Glucosyltransferase, Enzyme promiscuity, Flavonoid 7-*O*-Glycoside, Co-production, Metabolic engineering

## Abstract

Flavonoid 7-*O*-glycosides are a key class of bioactive flavonoid derivatives with broad application prospects in functional foods and nutraceuticals. Currently, their production is mainly dependent on unsustainable plant extraction or inefficient chemical synthesis. Microbial synthesis provides a promising green alternative for producing such functional ingredients; however, enzyme promiscuity hinders their ability to form specific products exclusively. To address this issue, we propose a strategy to harness enzyme promiscuity by engineering the metabolic context. This approach purposefully utilizes enzyme's catalytic flexibility to co-produce flavonoid 7-*O*-glycoside mixtures. We first optimized the (2*S*)-naringenin (NAR) biosynthesis module by overexpressing key enzymes, relieving feedback inhibition, and enhancing acetyl-CoA supply, achieving a yield of 318.08 mg/L NAR. Subsequently, by harnessing the promiscuity of 7-*O*-glucosyltransferase and implementing strategies including glycosidase elimination, *S*-adenosyl-l-methionine balancing, and uridine diphosphate-glucose supply optimization, we achieved efficient co-production of (2*S*)-isosakuranetin (ISOEIN) 7-*O*-glycoside and NAR 7-*O*-glycoside. This study establishes a sustainable and efficient biosynthesis platform for the production of complex flavonoid mixtures as potential functional food ingredients, demonstrates a green biosynthesis route for food-grade natural products, and exemplifies a novel paradigm of exploiting enzyme promiscuity through metabolic context engineering in microbial systems. This strategy is expected to be extendable to the synthesis of other structurally similar bioactive compounds for food and health applications.

## Graphical abstract

**Figure:**
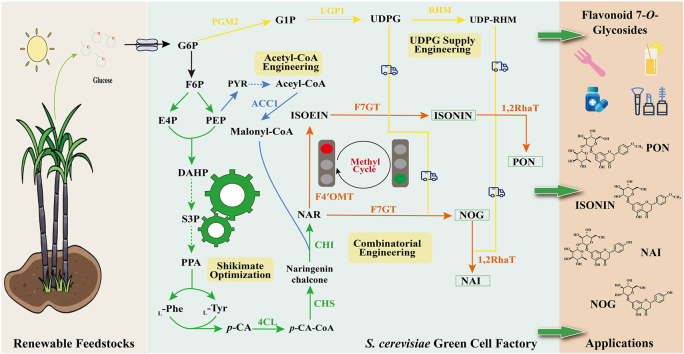

## 1 Introduction

Flavonoids, a prominent class of dietary polyphenols, are widely utilized in nutraceutical and pharmaceutical products owing to their diverse health-promoting activities [[1], [2], [3]]. Among them, flavonoid 7-*O*-glycosides demonstrate enhanced bioactivity, superior chemical stability, and improved oral bioavailability compared to their aglycone counterparts [4,5]. Representative compounds such as (2*S*)-isosakuranin (ISONIN), (2*S*)-naringenin 7-*O*-glucoside (NOG), (2*S*)-poncirin (PON), and (2*S*)-naringin (NAI) are primarily derived from *Poncirus trifoliata* and exhibit notable anti-inflammatory and antioxidant properties [[6], [7], [8]]. Beyond their physiological benefits, these compounds also show significant potential in food applications. For instance, microencapsulated NAI has been shown to reduce whey separation in yogurt and slow pH decline, thereby extending product shelf life—a characteristic that renders these glycosides highly attractive for functional food development [9].

However, the commercial supply of these valuable compounds still relies heavily on plant extraction, a process limited by seasonal variability and low efficiency [10,11]. Although chemical synthesis offers an alternative, it often requires expensive precursors, energy-intensive conditions, and complex protection/deprotection steps. Moreover, achieving stereochemical control at the C2 position of rhamnose—essential for synthesizing PON and NAI—remains particularly challenging [12,13]. These limitations underscore the urgent need for sustainable and scalable production strategies.

**Fig. 1:**
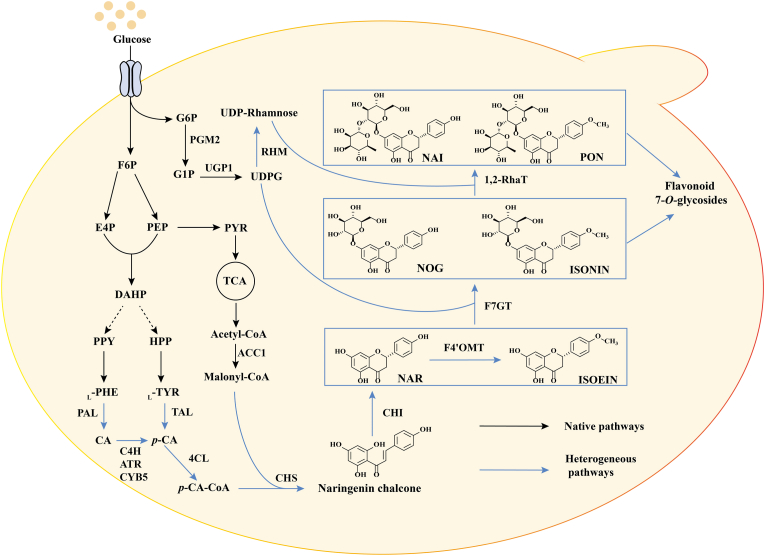
Engineered biosynthetic pathway for ISONIN, PON, NOG, and NAI in *S. cerevisiae*. Key metabolites and enzymes are abbreviated: G6P, glucose-6-phosphate; G1P, glucose-1-phosphate; F6P, fructose-6-phosphate; E4P, erythrose-4-phosphate; PEP, phosphoenolpyruvate; DAHP, 3-deoxy-arabino heptulonate-7-phosphate; PPY, phenylpyruvate; HPP, para-hydroxy-phenylpyruvate; l-TYR, l-tyrosine; l-PHE, l-phenylalanine; UDPG, UDP-glucose; NAR, (2*S*)-naringenin; ISOEIN, (2*S*)-isosakuranetin; NOG, (2*S*)-naringenin 7-*O*-glucoside; ISONIN, (2*S*)-isosakuranin; NAI, (2*S*)-naringin; PON, (2*S*)-poncirin; PGM2, phosphoglucomutase; UGP1, uridylyltransferase; TAL, tyrosine ammonia lyase; PAL, phenylalanine ammonia lyase; C4H, cinnamic acid hydroxylase; ATR, P450 reductase; CYB5, yeast native cytochrome *b*5; 4CL, 4-coumarate-CoA ligase; CHS, chalcone synthase; CHI, chalcone isomerase; F4′OMT, flavonoid 4′-*O*-methyltransferase; F7GT, flavonoid 7-*O*-glucosyltransferase; 1,2-RhaT, 1,2-rhamnosyltransferase; and RHM, UDP-rhamnose synthase.

Microbial biosynthesis in engineered *Saccharomyces cerevisiae* has emerged as a promising solution, capitalizing on the yeast's innate capacity to support cytochrome P450 enzymes involved in flavonoid biosynthesis [14,15]. The biosynthesis of ISONIN, PON, NOG, and NAI can be divided into two modules. The first is the (2*S*)-naringenin (NAR) synthesis module, converting aromatic amino acids (l-tyrosine/l-phenylalanine) to *p*-coumaric acid (*p*-CA) via enzymes including tyrosine ammonia lyase (TAL)/phenylalanine ammonia lyase (PAL) and cinnamate 4-hydroxylase (C4H), followed by *p*-coumarate-CoA ligase (4CL), chalcone synthase (CHS), and chalcone isomerase (CHI), to produce NAR [16,17]. The second is the post-modification module, involving sequential 4′-*O*-methylation by 4′-*O*-methyltransferase (F4′OMT), 7-*O*-glucosylation by 7-*O*-glucosyltransferase (F7GT), and 1,2-rhamnosylation by 1,2-rhamnosyltransferase (1,2-RhaT) [18,19]. Specifically, F7GT glycosylates NAR to yield NOG, while the combined action of F4′OMT and F7GT leads to ISONIN. These intermediates are then rhamnosylated by 1,2-RhaT to form NAI and PON, respectively (Fig. 1).

A critical hurdle in engineering these pathways is the intrinsic substrate promiscuity of the enzymes, which often compromises metabolic specificity. For example, expression of a *Mentha × piperita* 4′-*O*-methyltransferase (MpOMT) in *Escherichia coli* aimed at producing (2*S*)-hesperetin (HES) also generated substantial (2*S*)-isosakuranetin (ISOEIN) (1.86 mg/L HES vs. 9.65 mg/L ISOEIN) [18]. This promiscuity was even more pronounced in *Yarrowia lipolytica*, yielding 9.7 mg/L HES and 109.5 mg/L ISOEIN [20]. Similarly, expression of a flavonoid 7-*O*-glucuronosyltransferase in *Y. lipolytica* produced not only the target product baicalein (703.01 mg/L) but also apigenin 7-*O*-glucuronide (146.78 mg/L) [21]. Glycosyltransferase promiscuity is also observed in *S*. *cerevisiae*, as seen in anthocyanin biosynthesis, where levels of pelargonidin 3-*O*-glucoside (0.001 μmol/g CDW) were vastly different from those of kaempferol 3-*O*-glucoside (0.25 μmol/g CDW) [22]. Such enzyme promiscuity commonly leads to unintended byproduct accumulation, complicating the optimization of specific metabolite titers.

Rather than treating enzyme promiscuity as an obstacle to be eliminated, we propose to harness it through deliberate control of the metabolic context. Conventional metabolic engineering often seeks to suppress enzyme promiscuity to achieve single-product specificity—a process that is both metabolically costly and technically demanding. In contrast, we hypothesize that this inherent catalytic flexibility can be strategically harnessed. Inspired by the synergistic bioactivities of plant-derived flavonoid mixtures [23,24], we postulate that leveraging the inherent substrate promiscuity of F7GT can simplify pathway design, thereby alleviating the associated metabolic burden. Compared with conventional strategies that rely on multiple rounds of genetic editing to domesticate the enzyme for achieving ultra-high yield of a single product, directly harnessing its native promiscuity for the coordinated synthesis of multiple flavonoid 7-*O*-glycosides offers a mechanistically distinct advantage. This strategy avoids excessive and targeted intervention into the host metabolic network, minimizes disturbances to cellular homeostasis, and consequently circumvents the metabolic burden and fitness costs often associated with enforcing strict product specificity. Furthermore, it provides a more robust and sustainable route for the efficient production of flavonoid glycoside mixtures with potential synergistic therapeutic value. Therefore, the primary objective of this study was to develop an *S. cerevisiae* platform that directs metabolic flux through promiscuous enzymes to achieve balanced synthesis of these target flavonoid glycosides. By demonstrating the feasibility of this approach, we aim to establish a sustainable and transformative framework for the biosynthesis of complex natural product mixtures, thereby providing bioactive compounds for applications in the food, nutraceutical, and cosmetic industries.

## 2 Materials and methods

### 2.1 Chemicals and reagents

NAR, NOG, and NAI were purchased from Shanghai Macklin Biochemical Technology Co., Ltd. (Shanghai, China). ISOEIN, ISONIN, and PON were obtained from ChemFaces Biochemical Co., Ltd. (Wuhan, China). The Phanta Max Master Mix (Dye Plus), 2 × Rapid Taq Plus Master Mix (Dye Plus), one-step cloning kit, DNA gel extraction kit, and plasmid mini extraction kit were purchased from Nanjing Vazyme Biotechnology Co., Ltd. (Nanjing, China). Tryptone and yeast extract were purchased from Oxoid Co., Ltd. (Hampshire, UK). The 20 × yeast nitrogen base (YNB) medium was purchased from Sangon Bioengineering Co., Ltd. (Shanghai, China). Ampicillin was sourced from Solarbio Technology Co., Ltd. (Beijing, China). The primers and codon-optimized exogenous gene sequences were synthesized by Sangon Bioengineering Co., Ltd. (Shanghai, China).

### 2.2 Genetic manipulation

The *S. cerevisiae* CEN.PK2-1D-derivative strain SQ05 was used as the parent strain for all the engineering work. All endogenous genes, promoters, and terminators were amplified from the genomic DNA of CEN.PK2-1D-*ΔGAL80*, and all codon-optimized heterologous genes were amplified from synthetic fragments. *E. coli* JM109 was used for plasmid amplification. Overlap PCR was employed to obtain the donor DNA fragment. Gibson assembly was used for the construction of plasmids.

CRISPR/Cas9-mediated genome engineering was used for chromosomal gene deletion and the integration of expression cassettes. Yeast transformation was performed using the LiAc/SS carrier DNA/PEG method to introduce recombinant plasmids, integration, or knockout cassettes into *S. cerevisiae* [19]. Transformants were selected on YNB medium supplemented with appropriate amino acids and incubated at 30 °C for 3–5 days. Clones were confirmed by colony PCR [19]. Detailed descriptions of the genotypes of the primers (Table S1), DNA sequences (Table S2), plasmids (Table S3), gRNAs (Table S4), and strains (Table S5) are included in Supporting Information.

### 2.3 Cultivation and fermentation conditions

The Luria–Bertani (LB) medium used contained 10 g/L tryptone, 5 g/L yeast extract, 10 g/L NaCl, and 100 mg/L ampicillin. The yeast extract peptone dextrose (YPD) medium used contained 20 g/L tryptone, 10 g/L yeast extract, and 20 g/L glucose. The YNB medium contained 20 g/L glucose, 6.7 g/L YNB medium without amino acids, and the corresponding amino acids (50 mg/L leucine, 50 mg/L histidine, 50 mg/L tryptophan, or 50 mg/L uracil, depending on the corresponding screening label selected). The YPD-5-fluoroorotic acid (YPD-5-FoA) medium contained 20 g/L tryptone, 10 g/L yeast extract, 20 g/L glucose, 1 g/L 5-fluoroorotic acid, and 2% agar.

The selected seed culture, comprising a single clone on YPD-agar or YNB-agar, was transferred to 5 mL of YPD medium or YNB medium in 50 mL shake flasks and incubated at 30 °C and 220 rpm for 16 h (YPD medium) or 24 h (YNB medium). Subsequently, the cultures were inoculated at a concentration of 1% (*v/v*) in 25 mL of YPD medium in 250 mL shake flasks at 30 °C and 220 rpm for 96 h.

### 2.4 Metabolite extraction and quantification analysis

Metabolite extraction and quantification analyses were performed in line with our previous studies, with only minor modifications. For the quantification of synthetic flavonoids and their intermediates, samples (0.5 mL) were mixed with an equal volume of methanol and vortexed for 30 min. After centrifugation at 12,000×*g* for 2 min, the supernatant was filtered through a 0.22 μm membrane and analyzed by high-performance liquid chromatography with a diode array detector (HPLC-DAD; LC-20A; Shimadzu, Japan) using a reversed-phase C18 column (4.6 × 250 mm, 5 μm; Shimadzu). Separation was achieved with a gradient of solvent A (0.1% formic acid in water) and solvent B (acetonitrile) at 1 mL/min. The column temperature was maintained at 30 °C, and detection was performed at 290 nm. The gradient program was as follows: 0–1.5 min, 90% A, 10% B; 1.5–3.0 min, 90–70% A, 10–30% B; 3.0–30 min, 70–45% A, 30–55% B; 30–33 min, 45–90% A, 55–10% B; and 33–40 min, 90% A, 10% B. NAI, NOG, PON, ISONIN, NAR, and ISOEIN were detected at 290 nm with retention times of 10.6, 11.5, 14.5, 16.5, 20.5, and 30.5 min, respectively. The quantification of the aforementioned compounds was performed based on calibration curves derived from their corresponding reference standards.

### 2.5 Statistical analysis

The results are shown as the means ± standard errors of the mean deviation from three replicates. Statistical analyses were performed using SPSS 16.0.1 software (SPSS Inc., Chicago, IL, USA). One-way analysis of variance and independent-sample *t*-tests were employed to compare differences between samples. The original graphs were generated using Origin 2021 software (Massachusetts, USA).

## 3 Results and discussion

### 3.1 Combinatorial pathway regulation enables high-yield NAR production

**Fig. 2:**
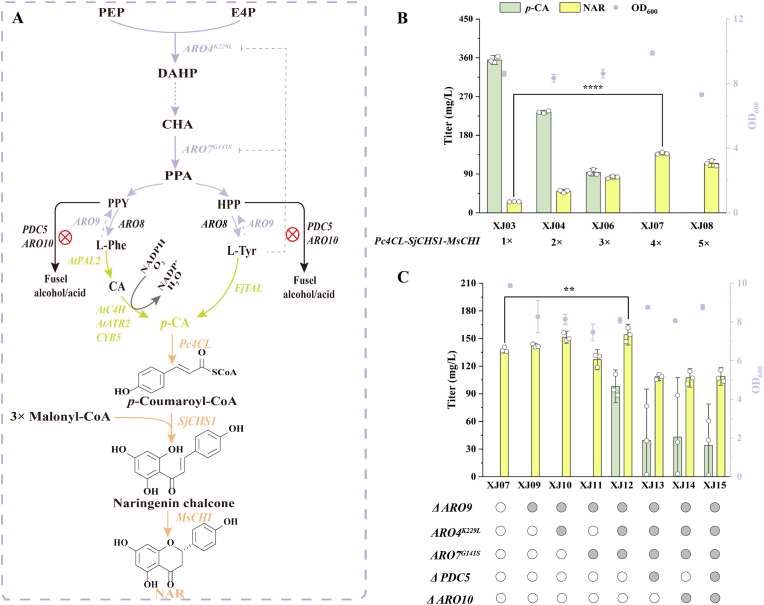
A synergistic metabolic engineering strategy for high-yield NAR production. (A) Schematic of the engineered NAR biosynthetic pathway. The upstream aromatic amino acid (AAA) biosynthetic pathway was enhanced (purple arrows), while competing branch pathways were blocked (red T-bars). The heterologous NAR synthesis pathway is shown with orange arrows. (B) NAR production was improved by increasing the copy number of genes in the heterologous pathway. (C) NAR titer was further enhanced by relieving a bottleneck in the AAA pathway and eliminating a degradation route for AAAs. Data are presented as mean ± SD (n = 3). Statistical significance was determined by Student's *t*-test: ∗∗*p* < 0.01, ∗∗∗∗*p* < 0.0001.

We engineered *S. cerevisiae* for the production of NAR, a central flavonoid precursor, by reconstructing the relevant pathways (Fig. 2A) [[25], [26], [27]]. The initial engineered strain XJ03, constructed by integrating *Pc4CL* (from *Petroselinum crispum*), *SjCHS1* (from *Sophora japonica*), and *MsCHI* (from *Medicago sativa*) into the *X3* locus of the *p*-CA producing strain SQ05 [15,28], produced only 25.56 mg/L NAR while accumulating 354.89 mg/L *p*-CA (Fig. 2B). The inefficiency indicated a bottleneck in the pathway from *p*-CA to NAR. To address this, we systematically optimized the gene dosage of the heterologous enzymes by integrating their expression cassettes into different chromosomal loci (specifically, the *XI3*, *XII1*, *XII5*, and *ARO10* genomic sites). This strategy revealed a distinct productivity threshold: strain XJ07 (harboring three additional gene copies) boosted NAR production to 137.78 mg/L, while strain XJ08 (derived from XJ07 with further gene amplification at the *ARO10* locus) showed a 25.87% reduction in growth, accompanied by a decrease in NAR titer to 114.60 mg/L (Fig. 2B). Although *ARO10* replacement could theoretically impaired growth, literature indicates that its deletion does not affect growth in flavonoid-producing strains [16,29]. This conclusion was further supported by our subsequent experiments: strain XJ14 (obtained by knocking out *ARO10* in strain XJ12) showed no significant growth difference compared to XJ12. Therefore, the observed growth inhibition is attributed not to the integration site itself, but to the metabolic burden resulting from overexpression of the NAR biosynthetic pathway genes. These results underscore the critical trade-off between metabolic burden and pathway yield.

The observed non-stoichiometric relationship between *p*-CA depletion and NAR accumulation can be attributed to several inherent challenges in pathway engineering. First, the diversion of *p*-CA by endogenous reductases such as *ADH6* and *ADH7* toward alternative products (e.g., *p*-coumaryl alcohol). Second, chalcone synthase (CHS) exhibits catalytic promiscuity and can catalyze non-productive side reactions, potentially generating byproducts such as coumaroyl triacetic acid lactone. Third, the high demand for malonyl-CoA (three molecules per NAR), whose competition with essential pathways like fatty acid biosynthesis can constrain CHS activity, leading to accumulation of upstream intermediates. Together, these factors collectively explain the carbon-flux discrepancy and highlight key targets for future pathway optimization.

To redirect the carbon flux from the shikimate pathway toward NAR synthesis, we engineered XJ07 by knocking out aromatic amino acid aminotransferase gene (*ARO9*) and overexpressing the feedback-insensitive mutants 3-deoxy-d-arabinoheptulosonate 7-phosphate synthase (*ARO4*^*K229L*^) and chorismate mutase (*ARO7*^*G141S*^) [30]. By combining these strategies in XJ07, we generated strains XJ09–XJ12. Among these, the strain XJ12 (overexpressing *ARO4*^*K229L*^ and *ARO7*^*G141S*^ at the *ARO9* locus) demonstrated a significant improvement, achieving a NAR yield of 154.20 mg/L and a *p*-CA yield of 98.01 mg/L (Fig. 2C). Strain XJ12 unlocked a substantial metabolic flux toward *p*-CA through these combinatorial modifications. These results demonstrate that relieving feedback inhibition and reducing aromatic amino acid (AAA) catabolism can significantly increase carbon flux toward NAR biosynthesis. Attempts to reduce AAA degradation by deleting *PDC5* or *ARO10* (pyruvate/phenylpyruvate decarboxylases) yielded strains XJ13–XJ15 with significantly lower NAR titers (Fig. 2C), confirming that the beneficial effects were pathway-specific.

### 3.2 Synthetic citrate valve-driven carbon shuttling boosts NAR biosynthesis via compartment-bridging catalysis

**Fig. 3:**
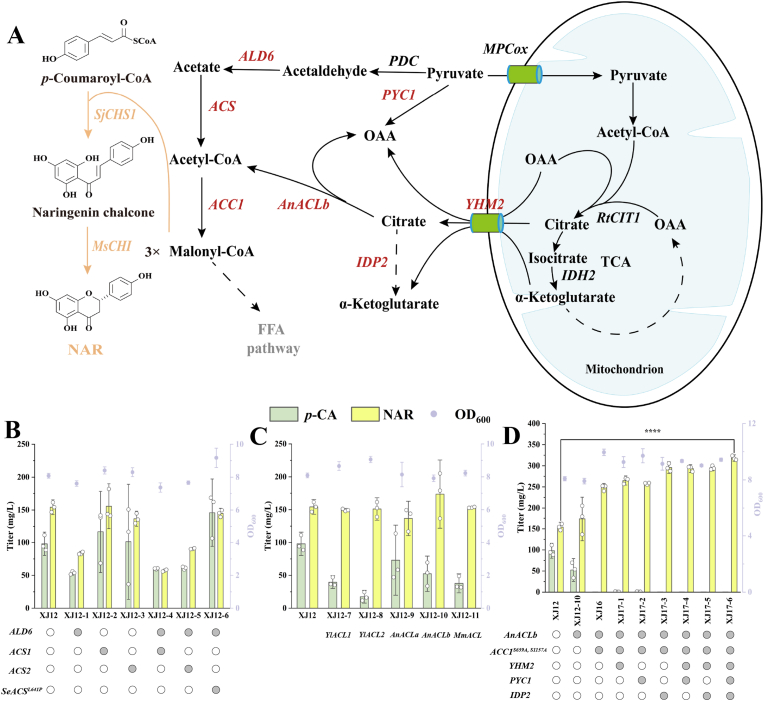
Metabolic engineering strategies to enhance NAR production by boosting cytosolic acetyl-CoA supply. (A) Schematic of acetyl-CoA subcellular trafficking. (B) Effects of improving endogenous cytosolic acetyl-CoA supply via the pyruvate dehydrogenase bypass pathway on NAR synthesis. Overexpressed genes are highlighted in red. (C) Expression of heterologous ATP-citrate lyase to link carbon trafficking between organelles and the cytosol. (D) Enhanced citrate synthesis and transport capacity promoted NAR overproduction. Data are presented as mean ± SD (n = 3). Statistical significance was determined by Student's *t*-test: ∗∗∗∗*p* < 0.0001.

NAR biosynthesis consumes one molecule of *p*-CA and three molecules of malonyl-CoA, which is derived from cytosolic acetyl-CoA [17,31]. Since acetyl-CoA is metabolically compartmentalized in *S. cerevisiae* (across the nucleus, cytoplasm, mitochondria, and peroxisomes) without direct intercompartmental transport [[32], [33], [34]], we targeted the cytosolic acetyl-CoA pool to drive NAR production. Our initial strategy involved overexpressing key genes of the pyruvate dehydrogenase bypass (*ALD6*, *ACS1*, and *ACS2* from *S. cerevisiae* and *SeACS*^*L641P*^ from *Salmonella enterica*) at the *X4* locus of the parental strain XJ12, thereby generating the engineered strains XJ12-1–XJ12-6 (Fig. 3A) [35]. Contrary to expectations, this not only failed to increase NAR but also significantly reduced titers in several strains (XJ12-1, XJ12-4, and XJ12-5; Fig. 3B), correlating with growth impairment (*ALD6*-overexpressing strains), confirming endogenous pathway toxicity [36].

We next engineered mitochondrial-to-cytosolic acetyl-CoA flux via a citrate export (Fig. 3A) [16,37]. We introduced ATP citrate lyase (ACL) from different sources—*YlACL1* and *YlACL2* from *Y. lipolytica*, *AnACLa* and *AnACLb* from *Aspergillus nidulans*, and *MmACL* from *Mus musculus*—into the *DPP1* locus of the strain XJ12, resulting in strains XJ12-7–XJ12-11, respectively. Among these, strain XJ12-10 achieved the highest NAR titer of 173.62 mg/L, a 12.59% increase compared to that in strain XJ12 (Fig. 3C). Subsequent expression of a deregulated acetyl-CoA carboxylase variant (*ACC1*^*S659A, S1157A*^) at the *X2* locus in this background yielded strain XJ16, which produced 248.00 mg/L NAR, a 42.84% improvement over that in strain XJ12-10.

To counter glucose-mediated citrate transport inhibition, we further overexpressed the mitochondrial carrier *YHM2*, pyruvate carboxylase *PYC1*, and NADP^+^-dependent isocitrate dehydrogenase *IDP2*, either individually or in combination, at the *X4* locus in XJ16 (XJ17-1–XJ17-6). All modifications increased NAR titers, with strain XJ17-6 achieving the highest titer of 318.08 mg/L, representing a 106.26% improvement over that in strain XJ12 (Fig. 3D). This work establishes that orchestrated mitochondrial–cytosolic carbon shuttling, via a synthetic citrate valve and compartment-bridging catalysis, is essential for high-level NAR synthesis.

### 3.3 Stepwise synthesis of flavonoid 7-*O*-glycosides

**Fig. 4:**
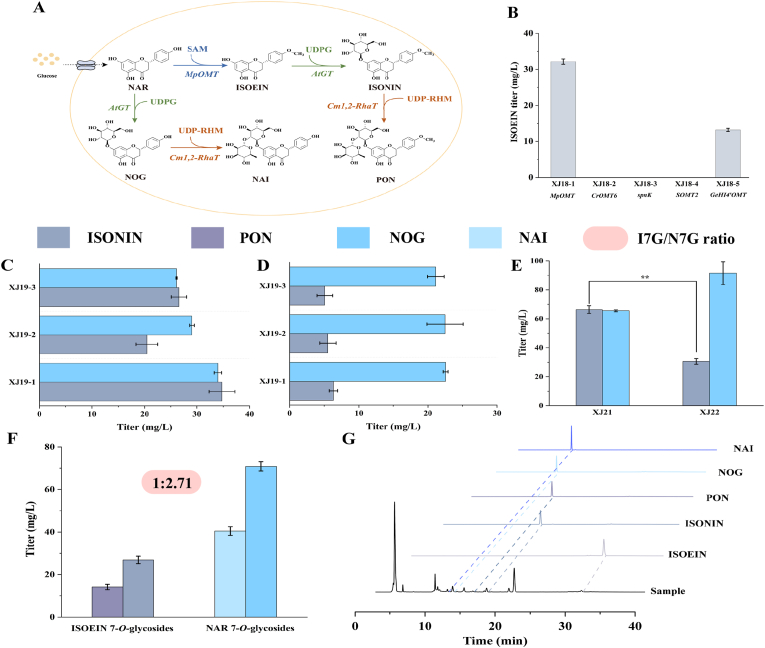
Biosynthesis of ISOEIN 7-*O*-glycosides and NAR 7-*O*-glycosides. (A) The stepwise biosynthetic pathway from glucose to the target compounds. I7G, ISOEIN 7-*O*-glycosides; N7G, NAR 7-*O*-glycosides. (B) Screening of F4′OMTs for ISOEIN synthesis. (C) Titers of ISONIN and NOG produced by AtGT in strains expressing *AtGT* under different promoter strengths at 48 h (D) and 96 h. The engineered strains were: XJ19-1 (pADH2-*AtGT*), XJ19-2 (pHXT7-*AtGT*), and XJ19-3 (pADH6-*AtGT*). (E) Titers of ISONIN and NOG in strains expressing *MpOMT* and *AtGT* via plasmid (XJ21) or genomic integration (XJ22) following the knockout of *EXG1*. (F) Titers of PON, ISONIN, NAI, and NOG in strain XJ23 after the introduction of *Cm1,*2-RhaT into strain XJ22. (G) Representative HPLC chromatograms of fermentation broth from strain XJ23 at 96 h, alongside authentic standards of PON, ISONIN, NAI, and NOG. Data are presented as mean ± SD (n = 3). Statistical significance was determined by Student's *t*-test: ∗∗*p* < 0.01.

Flavonoid 7-*O*-glycosides were prepared in this study using a stepwise synthesis method, in which F4′OMT, F7GT, and 1,2-RhaT are responsible for the post-modification steps (Fig. 4A). Five candidate F4′OMTs were first screened in strain XJ17-6 for the 4′-*O*-methylation modification of NAR: *MpOMT* from *Mentha × piperita*, *CrOMT6* from *Catharanthus roseus*, *spnK* from *Saccharopolyspora spinosa*, *SOMT2* from *Glycine max*, and *GeHI4′OMT* from *Glycyrrhiza echinata*. The strain XJ18-1 expressing *MpOMT* was selected for further pathway engineering, as it exhibited the highest ISOEIN titer (32.14 mg/L; Fig. 4B).

F7GT catalyzes the 7-*O*-glucosylation of flavonoids to yield flavonoid 7-*O*-glucosides. As a candidate for this reaction, we selected *AtGT* from *Arabidopsis thaliana*, which was recently reported to glycosylate the 7-OH position of flavonoids [19]. To optimize its expression, we constructed three strains (XJ19-1–XJ19-3) by expressing *AtGT* in the XJ18-1 background under the control of the pADH2, pHXT7, and pADH6 promoters, respectively. After 48 h, the ISONIN titers reached 34.76, 20.43, and 26.52 mg/L, demonstrating that the pADH2-driven system (strain XJ19-1) yielded the highest production (Fig. 4C). Notably, 34.00 mg/L of NOG was also detected in XJ19-1, indicating that AtGT can glycosylate both ISOEIN and NAR. The total titer of flavonoid 7-*O*-glucosides reached 68.76 mg/L, thus demonstrating the enzymatic basis for the concurrent biosynthesis of both ISONIN and NOG in a single fermentation process.

The total titer of flavonoid 7-*O*-glucosides decreased sharply at 96 h (28.88 mg/L; Fig. 4D), indicating potential degradation by endogenous glycoside hydrolases in *S. cerevisiae* [27,38,39]. According to previous studies, EXG1 is the primary endogenous hydrolase in *S. cerevisiae* responsible for hydrolyzing flavonoid glucosides [[40], [41], [42]]. Therefore, we hypothesize that the glucosidase EXG1 played a key role in this observed instability. To validate this, we generated an *EXG1*-knockout strain and reintroduced pPGK1-*MpOMT*-pADH2-*AtGT*, generating strain XJ21. Remarkably, this strain produced 131.97 mg/L of total flavonoid 7-*O*-glucosides at 96 h, a 3.57-fold increase over the control strain XJ19-1. This confirmed that knocking out *EXG1* not only prevented product degradation but also established the stability required for sustained cultivation. To address episomal plasmid instability for industrial applications, we chromosomally integrated the *MpOMT*-*AtGT* expression cassette into the *XII2* locus of the strain XJ20, resulting in strain XJ22. After 96 h, the ISONIN titer reached 30.60 mg/L, which was 1.17-fold lower than that of the plasmid-based strain XJ21 (Fig. 4E), likely owing to reduced expression efficiency in the integrated system compared to that in the high-copy plasmid system [43].

1,2-Rhamnosylation was the final step in the synthesis of flavonoid 7-*O*-neohesperidosides. We introduced the *Cm1,*2-RhaT gene from *Citrus maxima* into strain XJ22 at the *EXG1* locus. As this rhamnosylation requires UDP-rhamnose (UDP-RHM), a donor not natively produced by *S. cerevisiae*, we co-introduced *OlRHM* from *Ornithogalum longebracteatum* at the *LPP1* locus, thereby generating strain XJ23 [19,44]. After 96 h, strain XJ23 produced 41.04 mg/L of ISOEIN 7-*O*-glycosides (14.14 mg/L PON and 26.90 mg/L ISONIN) and 111.32 mg/L NAR 7-*O*-glycosides (40.46 mg/L NAI and 70.86 mg/L NOG), with a mass ratio of approximately 1:2.71 (Fig. 4F).

### 3.4 SAM precursor engineering reprograms MpOMT methylation selectivity to direct the flavonoid glycoside product ratio

**Fig. 5:**
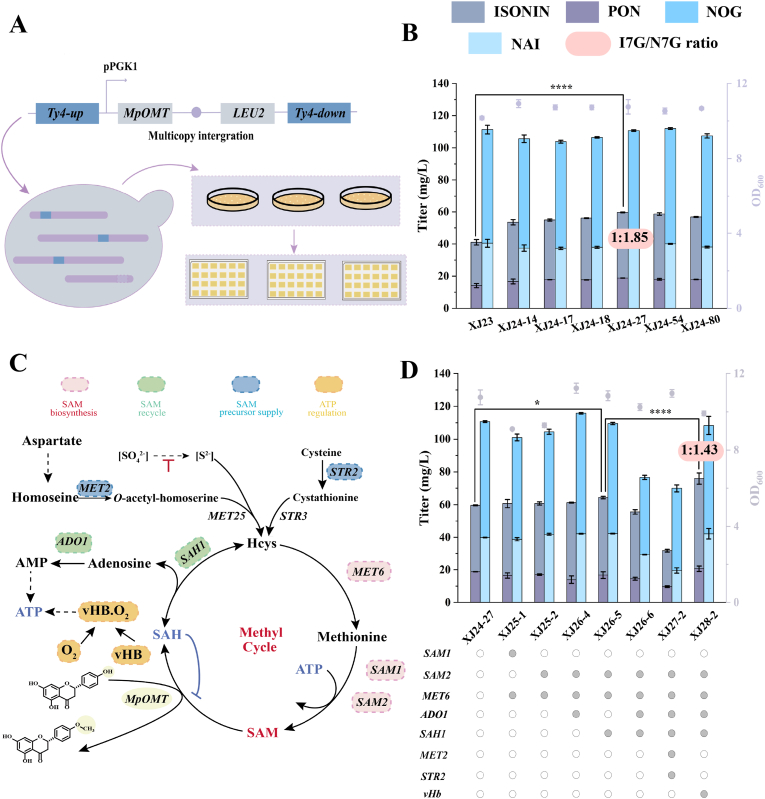
Reprogramming methylation selectivity for flavonoid 7-*O*-glycosides via optimized *MpOMT* expression and SAM precursor engineering. (A) Schematic of multicopy genomic integration of the *MpOMT* expression cassette. (B) Shake-flask screening to identify the strain with the highest production of ISOEIN 7-*O*-glycosides. (C) Schematic of the engineered SAM metabolism pathway. Dashed arrows represent multistep reactions, and double-headed arrows indicate reversible reactions. (D) Combined engineering of SAM biosynthesis, regeneration, and ATP supply improved both the titer of ISOEIN 7-*O*-glycosides and their ratio to NAR 7-*O*-glycosides. SAM1/SAM2, *S*-adenosyl-l-methionine synthetase; MET6, methionine synthase; ADO1, adenosine kinase; SAH1, SAH hydrolase; MET2, l-homoserine-*O*-acetyltransferase; STR2, cystathionine γ-synthase; vHb, *Vitreoscilla* hemoglobin; SAM, *S*-adenosyl-l-methionine; SAH, *S*-adenosyl-l-homocysteine; Hcys, l-homocysteine. Data are presented as mean ± SD (n = 3). Statistical significance was determined by Student's *t*-test: ∗*p* < 0.05, ∗∗∗∗*p* < 0.0001.

Strain XJ23 produced significantly lower titers of ISOEIN 7-*O*-glycosides than those of NAR 7-*O*-glycosides, suggesting a bottleneck at the 4′-*O*-methylation. To address this, we inserted a multicopy *MpOMT* expression cassette into the *Ty4* locus of strain XJ23 via chromosomal integration (Fig. 5A). The primary screening of 96 monoclonal colonies (strains XJ24-1–XJ24-96) in 24-well plates identified six strains (XJ24-14, -17, -18, -27, -54, and -80) with significantly elevated ISOEIN 7-*O*-glycoside production (Fig. S1). Subsequent validation in 250 mL shake flasks confirmed strain XJ24-27 as the top performer, achieving a titer of 59.66 mg/L ISOEIN 7-*O*-glycosides (a 45.37% increase compared to that in strain XJ23). These results demonstrate that *MpOMT* overexpression effectively alleviates this metabolic bottleneck, enhancing flux toward downstream methylated flavonoids.

In strain XJ24-27, the titer of NAR 7-*O*-glycosides (110.66 mg/L) was 1.85-fold higher than that of ISOEIN 7-*O*-glycosides, suggesting a persistent limitation of methyl donors (Fig. 5B). To reprogram the methylation selectivity of MpOMT, we engineered the availability of *S*-adenosyl-l-methionine (SAM) via four strategies (Fig. 5C): (1) Enhancing SAM biosynthesis by overexpressing *MET6* (synthesizes l-methionine from l-homocysteine [Hcys]) along with *SAM1*/*SAM2* (converts l-methionine to SAM) [45,46]; (2) Promoting SAM recycling by overexpressing SAH hydrolase (*SAH1*) to degrade *S*-adenosyl-l-homocysteine (SAH) into Hcys and adenosine, coupled with enhancing adenosine consumption via adenosine kinase (*ADO1*) to drive Hcys back into the methylation cycle [47,48]; (3) Increasing the supply of SAM precursors by overexpressing of *MET2* and *STR2*, the main rate-limiting genes in the Hcys production pathways from cysteine and aspartate [47]; and (4) Augmenting ATP supply by expressing *vHb* from *Vitreoscilla* hemoglobin to enhance oxygen-dependent ATP synthesis [49,50].

Overexpression of *MET6* and *SAM1* (XJ25-1) or *SAM2* (XJ25-2) in the *HO* locus of strain XJ24-27 yielded 60.69 mg/L and 60.75 mg/L of ISOEIN 7-*O*-glycosides, respectively, showing no significant improvement compared to the control yields (Fig. 5D). Despite the minimal change in glycoside yield, the intracellular ISOEIN titer increased by 20.38% to 25.93 mg/L in XJ25-1 and by 12.58% to 24.25 mg/L in XJ25-2 (from a baseline of 21.54 mg/L), suggesting an enhanced methylation flux from NAR. To further redirect flux toward ISOEIN 7-*O*-glycosides, we individually or combinatorially expressed *SAH1* and *ADO1* in the *XII3* locus of strain XJ25-2 to enhance SAM recycling. Individually expression of *SAH1* (XJ26-5) efficiently reprogrammed SAM utilization, elevating the ISOEIN 7-*O*-glycoside and ISOEIN titers to 64.37 mg/L and 30.28 mg/L, respectively. However, overexpression of the rate-limiting genes *MET2* and *STR2* at the *XII4* locus (XJ27-2) reduced the production of ISOEIN 7-*O*-glycoside to 31.61 mg/L (Fig. 5D), likely due to the disruption of cysteine/aspartate metabolism in *S. cerevisiae*.

The final optimization step involved integrating *vHb* into the *EGH1* locus of strain XJ26-5 to generate strain XJ28-2, which significantly enhanced SAM-driven selectivity. The strain achieved an ISOEIN 7-*O*-glycoside titer of 75.98 mg/L, an 18.04% increase that is attributable to enhanced oxygen utilization and ATP-dependent SAM synthesis [49]. This engineered SAM flux successfully reprogrammed the product selectivity of MpOMT, as evidenced by a decrease in the NAR 7-*O*-glycoside titer to 108.31 mg/L and a concurrent increase in the ISOEIN 7-*O*-glycoside titer. Consequently, the product ratio shifted from 1:1.85 (ISOEIN: NAR 7-*O*-glycoside in XJ24-27) to 1:1.43, representing a 54% reduction in the initial imbalance (Fig. 5D).

### 3.5 UDPG supply tuning redirects glycosyltransferase substrate selectivity for a balanced flavonoid glycoside output

**Fig. 6:**
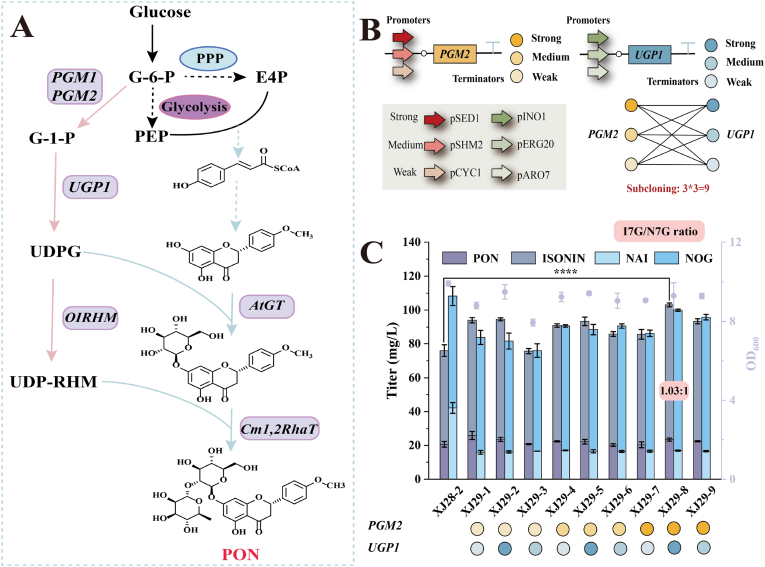
Engineering UDP-glucose (UDPG) supply to enhance flavonoid 7-*O*-glucoside production. (A) Biosynthetic pathway of PON and NAI from glucose, highlighting the roles of the glycosyl donors UDPG and UDP-rhamnose (UDP-RHM). (B) Schematic for modulating UDPG pathway gene expression using promoters of varying strengths. (C) Titers of flavonoid 7-*O*-glucosides in engineered strains with optimized UDPG pathway expression. Data are presented as mean ± SD (n = 3). Statistical significance was determined by Student's *t*-test: ∗∗∗∗*p* < 0.0001.

To further leverage enzyme promiscuity in directing the product ratio (ISOEIN: NAR 7-*O*-glycoside), we engineered the glycosylation pathway downstream. The biosynthesis of flavonoid 7-*O*-glucosides requires UDPG as the glucosyl donor, while their subsequent conversion to flavonoid 7-*O*-neohesperidosides depends on UDP-RHM [19,44]. This specialized nucleotide sugar is derived from UDPG via a heterologous trifunctional enzyme, RHM, through three sequential reactions involving UDP-4-keto-6-deoxyglucose and UDP-4-keto-l-rhamnose (Fig. 6A) [51]. Both glycosylation steps rely on UDPG availability, creating a tunable node for directing flux toward target glycosides.

In strain XJ28-2, we exploited glycosyltransferase promiscuity by modulating the UDPG supply to bias conversion toward ISOEIN 7-*O*-glucosides. Specifically, we overexpressed phosphoglucose mutase (*PGM2*) and UDP-glucose pyrophosphorylase (*UGP1*) at the *YPRCδ15C* locus using combinatorial promoter engineering to fine-tune the enzyme activity ratios. We employed a set of promoters with varying strengths: *PGM2* was driven by the strong (pSED1), medium (pSHM2), or weak (pCYC1) promoters, while *UGP1* was controlled by the strong (pINO1), medium (pERG20), or weak (pARO7) promoters. This approach generated nine combinatorial strains (XJ29-1–XJ29-9; Fig. 6B) [19].

Shake flask assays revealed distinct production profiles, with strain XJ29-8 (pSED1-*PGM2*; pINO1-*UGP1*) achieving the highest total titer of glycosylated derivatives (202.98 mg/L). The compounds synthesized by strain XJ29-8 were identified as ISONIN, NOG, PON, and NAI by comparison with standards using LC-MS (Fig. S2–S5). In this top-performing strain, the ratio between two major glucosylated products, ISOEIN 7-*O*-glucoside and NAR 7-*O*-glucoside, was successfully reprogrammed to 1.03:1 (103.10 mg/L vs. 99.88 mg/L; Fig. 6C), shifting it substantially closer to parity than the 1:1.43 ratio in strain XJ28-2. Critically, boosting the UDPG supply preferentially enhanced the flux toward the glucosylated products, demonstrating that substrate supply engineering can harness the inherent promiscuity of glycosyltransferases to rebalance product ratios toward a desired profile.

## 4 Conclusion

This study establishes a paradigm in *S. cerevisiae* to harness enzyme promiscuity for the targeted co-production of flavonoid 7-*O*-glycosides. This was achieved by systematically optimizing the NAR biosynthesis module and precisely regulating promiscuous glycosylation fluxes via the elimination of competing pathways and glycosidase activities, combined with a balanced supply of key cofactors. The engineered strain produced 202.98 mg/L of a bioactive mixture, comprising 103.10 mg/L of ISOEIN 7-*O*-glucoside and 99.88 mg/L of NAR 7-*O*-glucoside, achieving a nearly 1:1 ratio. This represents a significant improvement from the initial ratio of 1:2.71. The developed microbial platform demonstrates distinct advantages over conventional extraction or chemical synthesis approaches. The process is non-toxic, energy-efficient, and completes within only 4 days, free from seasonal or geographical constraints. Consequently, this strategy provides a sustainable, efficient, and versatile route to produce complex natural products for functional food ingredients.

## CRediT authorship contribution statement

**Xinjia Tan:** Writing – review & editing, Writing – original draft, Software, Methodology, Formal analysis, Data curation. **Shasha Zuo:** Methodology, Formal analysis, Data curation. **Fanglin Hu:** Methodology, Formal analysis. **Zhiqiang Xiao:** Methodology, Formal analysis. **Yongtong Wang:** Software, Formal analysis. **Siqi Zhang:** Formal analysis. **Qiyuan Lu:** Data curation. **Yifei Zhao:** Formal analysis. **Jiaxu Chen:** Data curation. **Liusha Fan:** Formal analysis. **Juan Liu:** Writing – review & editing, Supervision, Funding acquisition, Conceptualization. **Yang Shan:** Supervision, Project administration, Funding acquisition, Conceptualization.

## Abbreviations

NAR, (2*S*)-naringenin; ISOEIN, (2*S*)-isosakuranetin; ISONIN, (2*S*)-isosakuranin; NOG, (2*S*)-naringenin 7-*O*-glucoside; PON, (2*S*)-poncirin; NAI, (2*S*)-naringin; *p*-CA, *p*-coumaric acid; HES, (2*S*)-hesperetin; TAL, tyrosine ammonia lyase; PAL, phenylalanine ammonia lyase; C4H, cinnamate 4-hydroxylase; 4CL, *p*-coumarate-CoA ligase; CHS, chalcone synthase; CHI, chalcone isomerase; F4′OMT, 4′-*O*-methyltransferase; F7GT, 7-*O*-glucosyltransferase; 1,2-RhaT, 1,2-rhamnosyltransferase; MpOMT, *Mentha* × *piperita* 4′-*O*-methyltransferase; YNB, yeast nitrogen base; LB, Luria–Bertani; YPD, yeast extract peptone dextrose; YPD-5-FoA, YPD-5-fluoroorotic acid; AAA, aromatic amino acid.

## Declaration of competing interest

The authors declare that they have no known competing financial interests or personal relationships that could have appeared to influence the work reported in this paper.
