# Supplementary material for: Enzyme promiscuity-driven co-production of flavonoid 7-O-glycosides in engineered Saccharomyces cerevisiae
Source: Synth Syst Biotechnol. 2026 Feb 12;13:251–60. doi: 10.1016/j.synbio.2026.01.033 (PMC12925191; doi:10.1016/j.synbio.2026.01.033)
Supplement: Multimedia component 1 [file mmc1.docx]

**Supporting Information**

**Enzyme Promiscuity-Driven Co-production of Flavonoid 7-*O*-Glycosides in Engineered *Saccharomyces cerevisiae***

Xinjia Tan ^a b c^, Shasha Zuo ^a b c^, Fanglin Hu ^a b c^, Zhiqiang Xiao ^a b^, Yongtong Wang ^a b c^, Siqi Zhang ^a b c^, Qiyuan Lu ^a b c^, Yifei Zhao ^a b c^, Jiaxu Chen ^a b d^, Liusha Fan ^a b c^, Juan Liu ^a b *^, and Yang Shan ^a b c*^

^a^ Hunan Institute of Agricultural Products Processing and Quality Safety; Dongting Laboratory; Hunan Academy of Agricultural Sciences, Changsha 410125, China

^b^ Yuelushan Laboratory, Changsha 410128, China

^c^ Longping Agricultural College, Hunan University, Changsha 410125, China

^d^ College of Food Science and Nutritional Engineering, China Agricultural University, Beijing 100083, China

*Corresponding authors: Juan Liu, and Yang Shan

E-mail addresses: Juan Liu (liujmax2019@163.com); Yang Shan (sy6302@sohu.com)

**
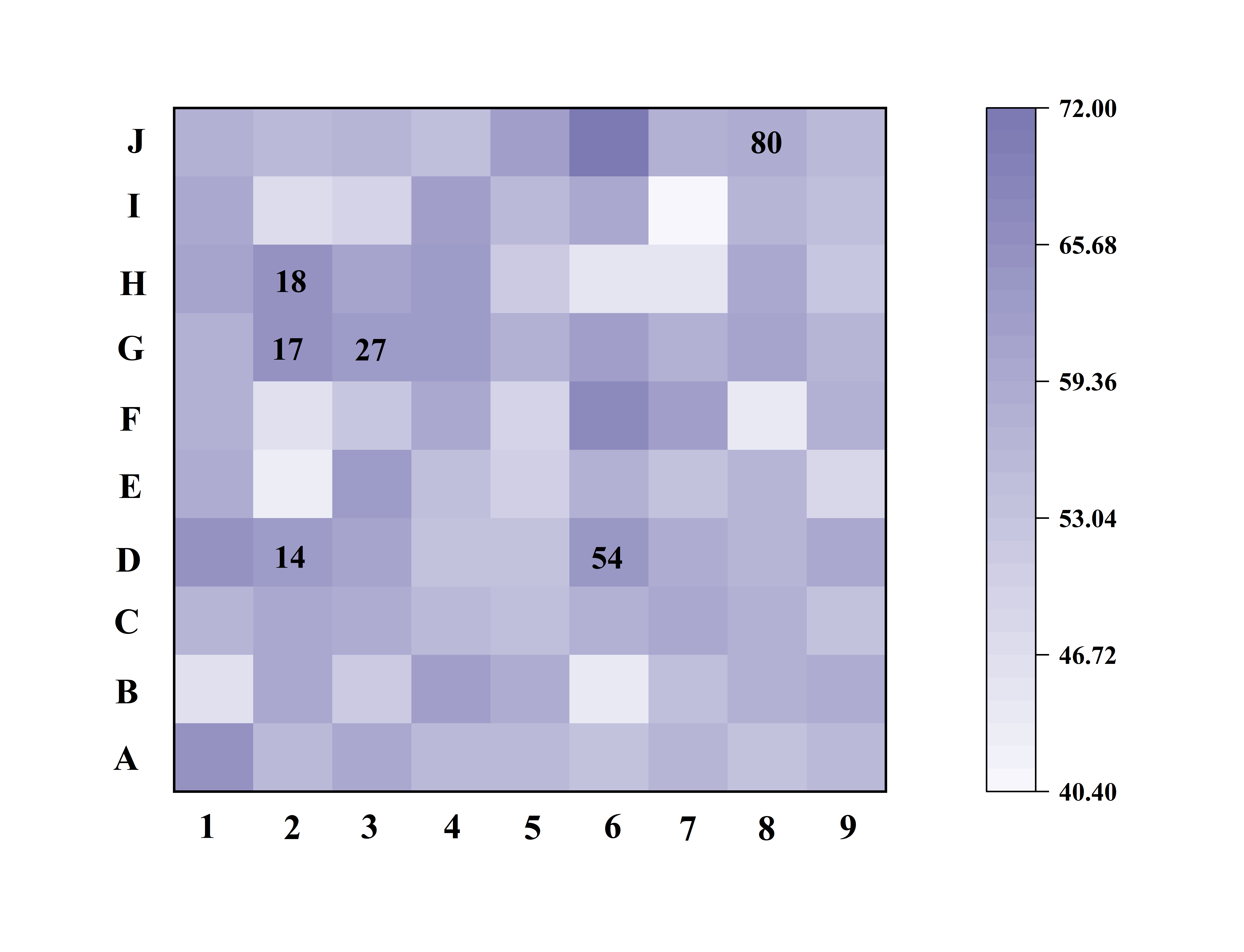
**

**Fig. S1.** Primary screening of yeast transformants obtained through multiple copy site integration in 24-deep-well plates.


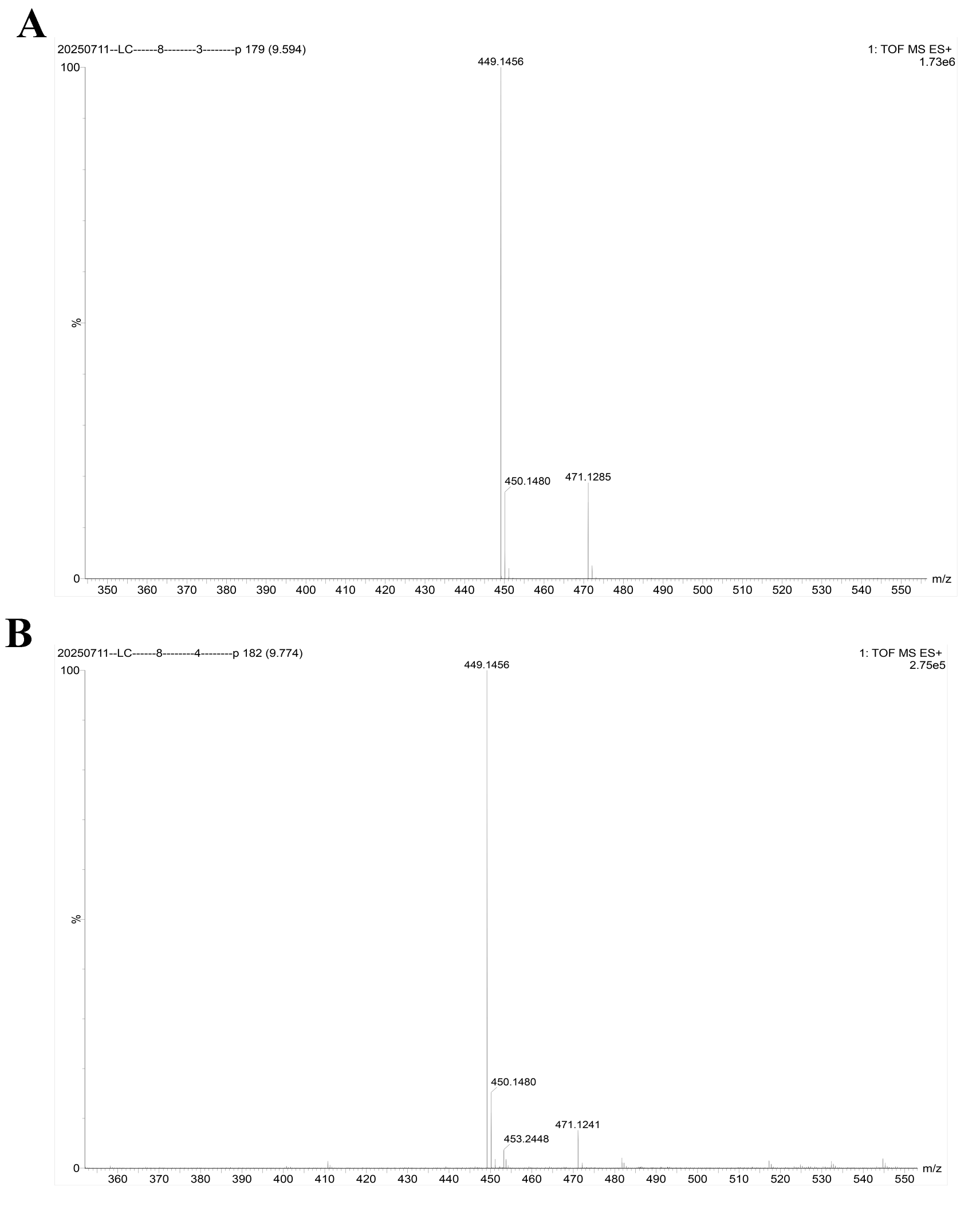


**Fig. S2.** (A) LC–MS results of the ISONIN standard. (B) LC–MS results of the fermentation broth from strain XJ29-8 at 96 h.


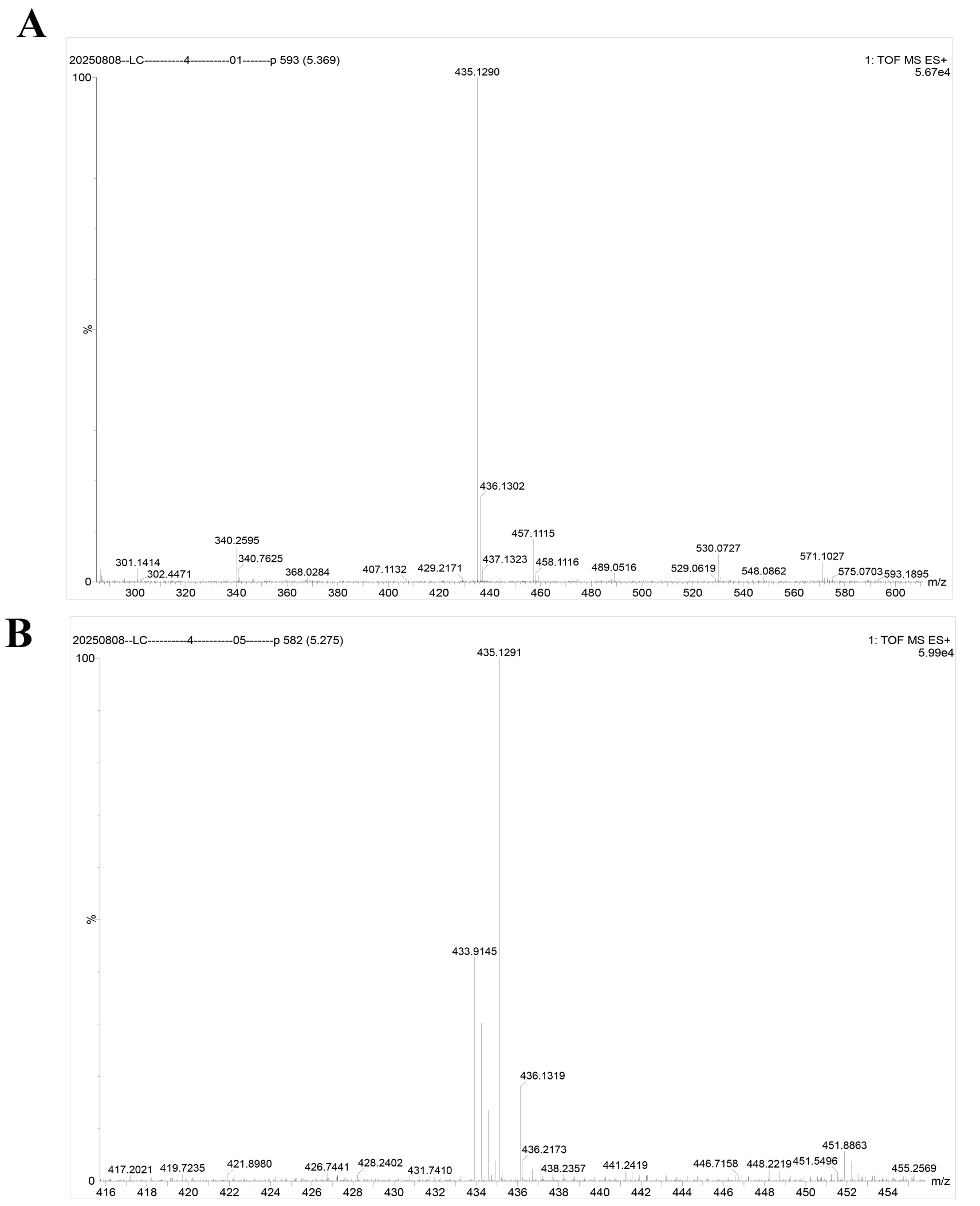


**Fig. S3.** (A) LC–MS results of the NOG standard. (B) LC–MS results of the fermentation broth from strain XJ29-8 at 96 h.


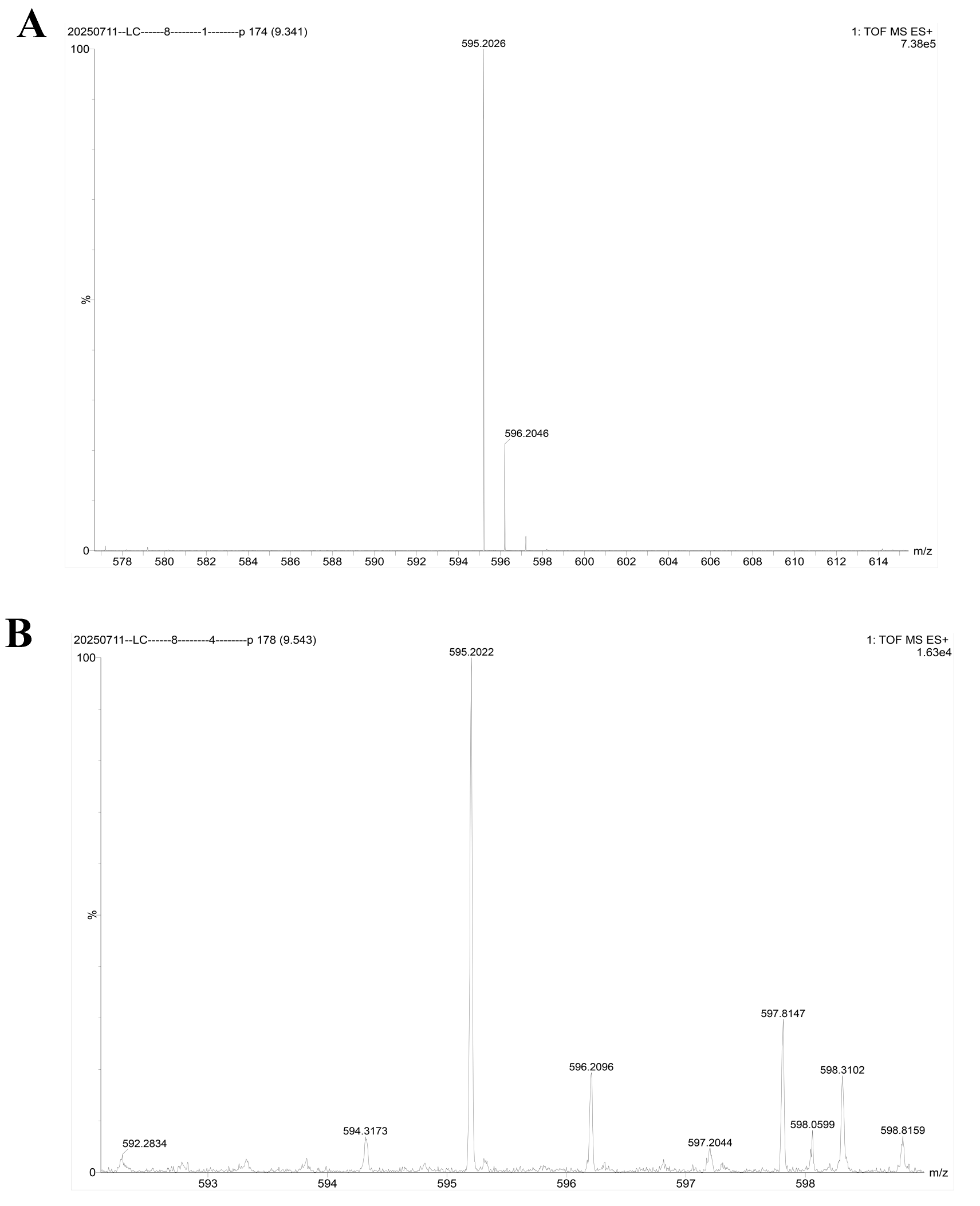


**Fig. S4.** (A) LC–MS results of the PON standard. (B) LC–MS results of the fermentation broth from strain XJ29-8 at 96 h.


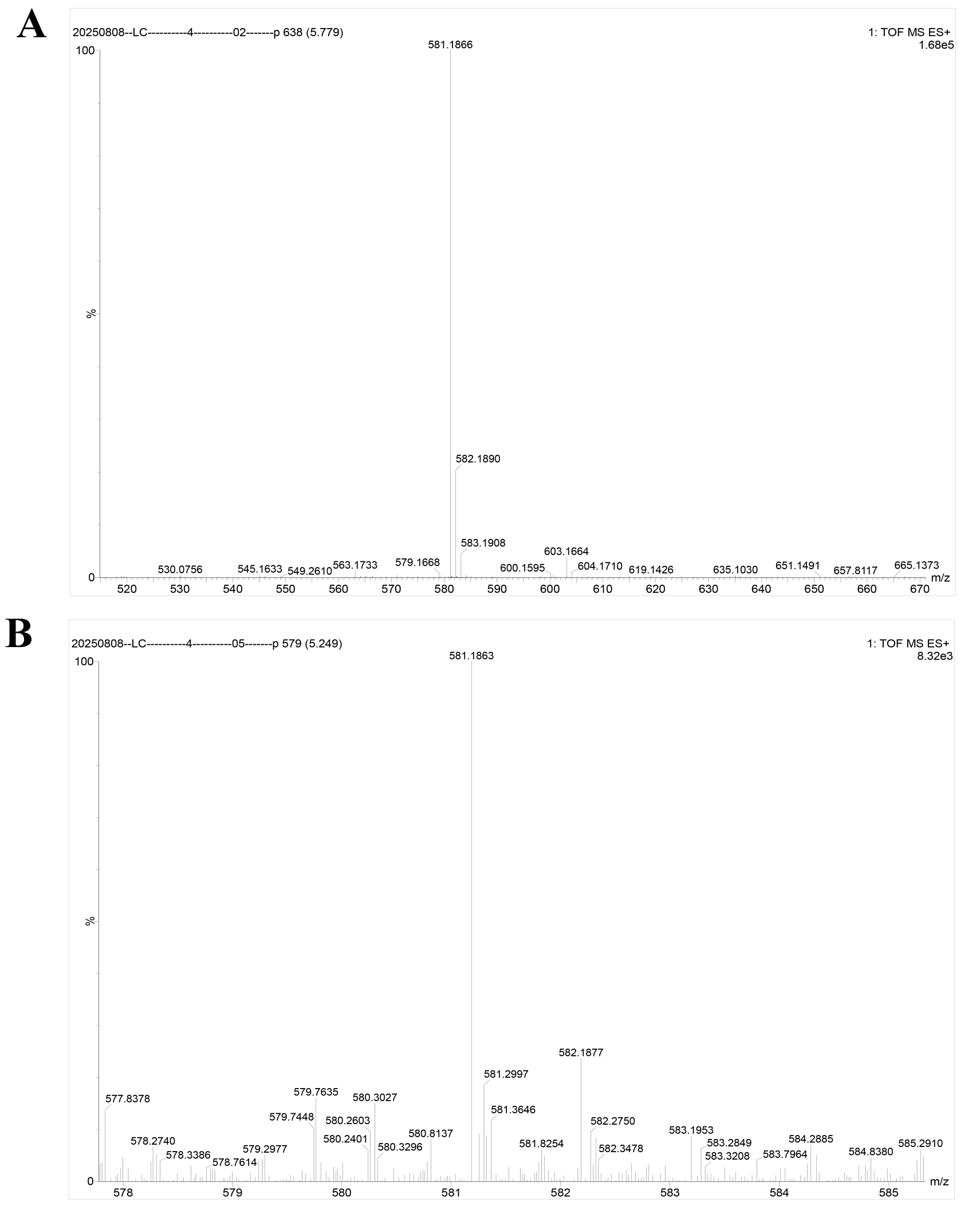


**Fig. S5.** (A) LC–MS results of the NAI standard. (B) LC–MS results of the fermentation broth from strain XJ29-8 at 96 h.

**Table S1. Primers used in this study.**

| **Primer** | **Sequence (5’–3’)** |
| --- | --- |
| Amplification of promoters, terminators, and genes for the synthesis of genistein. | |
| X3A-F | aaaggaactttcaccctacagcacc |
| X3A-R | GGTATAGCATGAGGTCGCTCcgtttctaaggccacttttcaatgaaacg |
| SjCHS1-ADH1t-F | GTTCCTTTGCAAGGTTAAGCGAATTTCTTATGATTTATGATTTTTATT |
| ADH1t-R | GAGCGACCTCATGCTATACCTGAGA |
| SjCHS1-F | ATGGTTACAGTTGAAGAAATTAGAAATG |
| SjCHS1-R | TTAACCTTGCAAAGGAACAGAATG |
| GAL1,10p-F | tatagttttttctccttgacgttaaagtatag |
| GAL1,10p-SjCHS1-R | CTTCAACTGTAACCATttatattgaattttcaaaaattcttacttttt |
| FBA1p-F | tactttaacgtcaaggagaaaaaactataATAACAATACTGACAGTACTAAATAATTGC |
| FBA1p-R | TTTGAATATGTATTACTTGGTTATGG |
| MsCHI-F | ACCATAACCAAGTAATACATATTCAAAatggcagcaagcattacggc |
| MsCHI-R | GACATAAGAGATCCGCtcagttaccgattttaaaggcaccttc |
| ADH2t-F | GCGGATCTCTTATGTCTTTACGATTTATAGTTTTC |
| ADH2t-R | AATCAGTGGTGTATGGAAGGTAGAATGAGAAATATCGAGGGAGACGA |
| SED1p-F | TCTACCTTCCATACACCACTGATTGCTC |
| SED1p-R | gtcacccatCTTAATAGAGCGAACGTATTTTATTTTGC |
| Pc4CL-F | c atgggtgactgcgttgcc |
| Pc4CL-R | ttacttcggcaggtcgcc |
| CYC1t-F | cctgccgaagtaatcatgtaattagttatgtcacgcttacattcacgc |
| CYC1t-R | gtaatacgactcactatagggcgaattgg |
| X3B-F | ccctatagtgagtcgtattaccgaaagatttctcttgcgatttcttcg |
| X3B-R | ccttctaataatttgtcatttgactgacgaatcg |
| XI3A-F | ttttttgcttttcccgttatgagaaatgttttttttag |
| XI3A-R | GTATAGCATGAGGTCGCTCgggctgatcattttccttacgtgg |
| XI3B-F | ccctatagtgagtcgtattacttcttttcttagaagaggttttgttaccg |
| XI3B-R | atttctgttattactgttattagtttggcgttttgc |
| XII1A-F | GTTGAGCTCTGTCCTTCATGGACT |
| XII1A-R | GTATAGCATGAGGTCGCTCGAAAGAACCGAACCGATGCCAAAAT |
| XII1B-F | ctatagtgagtcgtattacGTTCAGTTTAGTGCTCTGTCTGAGTGAC |
| XII1B-R | TGATGACTGTTTCTCAATCTTTATGTTATCTTCTTACACC |
| XII5A-F | GTAGTGATCATTGGCTTAACGAAACGG |
| XII5A-R | GTATAGCATGAGGTCGCTCGTGACAATAAATTCAAACCGGTTATAGCGG |
| XII5B-F | ctatagtgagtcgtattacCAACTCAGAAGTTTGACAGCAAGCAAG |
| XII5B-R | CTCTTTTGCCTTTCAAAAAAGGATTAAATAGAGTTAATC |
| ARO10A-F | GCTTGTACACCTCATGTAGCTTCCTTATTAAC |
| ARO10A-R | GCATGAGGTCGCTCGCTTAAGGGAGTTTCTTTGTTATCTTGTAAATAAACTTTAC |
| ARO10B-F | ctatagtgagtcgtattacaaactTGTGGGCGCAATTATAAAACAC |
| ARO10B-R | GCTCTATTACTTCCTGCTTTGTTAGAGCC |
| ARO9A-F | GTATGTTACAACAGTCAAAGTAATGAACTTTCGC |
| ARO9A-ARO9B-R | GGTTTAATCTTCACTGTTGAGTCGATGAGAGAGTGTAATTGTGGTATG |
| ARO9B-F | ACAGTGAAGATTAAACCTATGTGTTATTTGCATATC |
| ARO9B-R | CTCGTTATATTACAACATTTAAATCTATTGTGTAACAGACTATAGC |
| ARO9A-R | TGAGTCGATGAGAGAGTGTAATTGTGGTATG |
| ENO2p-F | CACTCTCTCATCGACTCACGACGTGCACCAACTTGC |
| ENO2p-R | tattattgtatGTTATAGTATTAGTTGCTTGGTGTTATGAAAG |
| ARO4(K229L)-F | CTATAACatacaataataATGAGTGAATCTCCAATGTTCGCTG |
| K229L-R | CAGAGTAACACCCATGAAATGGTGAG |
| K229L-F | TTCATGGGTGTTACTCTGCATGGTGTTGCTGCTATCAC G |
| ARO4(K229L)-R | CTATTTCTTGTTAACTTCTCTTCTTTGTCTGACAGC |
| IDP1t-F | GAAGTTAACAAGAAATAGTCGAATTTACGTAGCCCAATCTaccac |
| IDP1t(ARO9B)-R | GGTTTAATCTTCACTGTGACGTCAAAAGAAGTATTATATGAGCCTATGAAAGAAAT |
| FBA1p(ARO9A)-F | CACTCTCTCATCGACTCATGGGTCATTACGTAAATAATGATAGGAATGGG |
| FBA1p-R | TTTGAATATGTATTACTTGGTTATGGTTATATAtgacaaaagaaaaag |
| ARO7(G141S)-F | CAAGTAATACATATTCAAAATGGATTTCACAAAACCAGAAACTGTTTTAAATC |
| G141S-R | ACTGAAGTTATTCTTATCATCACCATCTCTTTTCG |
| G141S-F | GATAAGAATAACTTCAGTTCTGTTGCCACTAGAGATATAGAATGTTTG |
| ARO7(G141S)-R | TTACTCTTCCAACCTTCTTAGCAAGTATTCC |
| CYC1t-ARO7(G141S)-F | GAAGGTTGGAAGAGTAAACAGGCCCCTTTTCCTTTGT |
| CYC1t-ARO9B-R | GGTTTAATCTTCACTGTGCAAATTAAAGCCTTCGAGCGTC |
| IDP1t-R | GACGTCAAAAGAAGTATTATATGAGCCTATGAAAGAAAT |
| FBA1p-IDP1t-F | GGCTCATATAATACTTCTTTTGACGTCTGGGTCATTACGTAAATAATGATAGGAATGGG |
| PDC5A-F | GCCAAGGAAATAAAGcaaataacaataacacc |
| PDC5A-R | GAGTTTTATGTTAATTAGCtttgttcttcttgttaTTGTATTGTGTTGTTCTCTTTG |
| PDC5B-F | GCTAATTAACATAAAACTCATGATTCAACGTTTgtg |
| PDC5B-R | CTAAATAAGATGTAAGGCCTTGTAATTCAGTTTGTTcac |
| ARO10A-F | GCTTGTACACCTCATGTAGCTTCCTTATTAAC |
| ARO10A-R | GCTTAAGGGAGTTTCTTTGTTATCTTGTAAATAAACTTTAC |
| ARO10B-F | CAAAGAAACTCCCTTAAGCaaactTGTGGGCGCAATTATAAAACACa |
| ARO10B-R | GCTCTATTACTTCCTGCTTTGTTAGAGCC |
| X4A-F | cccaaagctaagagtcccattttattcttctatatgtata |
| X4A-TEF1p-R | GAAGCTATGGTGTGTGGctgctcttgaatggcgacagc |
| TEF1p-F | CCACACACCATAGCTTCAAAATGTTTC |
| TEF1p-R | TTTGTAATTAAAACTTAGATTAGATTGCTATGCTTTCTTTCTAATG |
| ALD6-TEF1p-F | CTAAGTTTTAATTACAAAATGACTAAGCTACACTTTGACACTGC |
| ALD6-TPS1t-R | GTCTCATTTGCATCGGGTTCATTACAACTTAATTCTGACAGCTTTTACTTCAGTG |
| TPS1t-F | TGAACCCGATGCAAATGAGACG |
| TPS1t-R | ttttgtcttcttcaaattgaaGTTACTTTCTAAAATGGC |
| X4B-TPS1t-F | atttgaagaagacaaaaaacaggcatgggaagattcgc |
| X4B-R | ctggtgaggatttacggtatgatcatg |
| PGK1p-X4A-F | gtcgccattcaagagcagGGGCCAGAAAAAGGAAGTGTTTCC |
| PGK1p-R | tgttttatatttgttGTAAAAAGTAGATAATTACTTCCTTGATGATC |
| ACS1-PGK1p-F | CaacaaatataaaacaATGTCGCCCTCTGCCG |
| ACS1-PGK1t-R | GATTTCAATTCAATTcaatTTACAACTTGACCGAATCAATTAGATGTCTAACAATG |
| PGK1t-F | attgAATTGAATTGAAATCGATagatcaatttttttcttttc |
| PGK1t-R | AACGCAGAATTTTCGAGTTATTAAACTTAAAATACG |
| X4B-PGK1t-F | CTCGAAAATTCTGCGTTaacaggcatgggaagattcgc |
| ACS2-PGK1t-F | CaacaaatataaaacaATGACAATCAAGGAACATAAAGTAGTTTATGAAGC |
| ACS2-PGK1t-R | TTTCAATTCAATTcaatTTATTTCTTTTTTTGAGAGAAAAATTGGTTCTCTACAGC |
| SSA1p-F | tatgtgtgtatatattcggttggactcttcagtg |
| SSA1p-R | attatctgttatttacttgaatttttgtttcttgtaatacttgattac |
| SeACSL641P-F | caagtaaataacagataatatgtcacaaactcataaacatgctattccag |
| SeACSL641P-R | atgaatggaattgctggcggccgcttaagatgg |
| TAT1t-F | cagcaattccattcattttccgcttg |
| TAT1t-R | ttctaaaatcacttgagttttattgaagctaaataaattgatagtatcg |
| X4B-TAT1t-F | ctcaagtgattttagaaaacaggcatgggaagattcgc |
| X4A-TDH3t-R | aaattccgctgtatagcctgctcttgaatggcgacagc |
| TDH3t-F | gctatacagcggaatttccatatcactcag |
| TDH3t-R | gtgaatttactttaaatcttgcatttaaataaattttctttttatagc |
| ALD6-ADH2p-F | ctatatcgtaatacacaATGACTAAGCTACACTTTGACACTGC |
| ALD6-TDH3t-R | gatttaaagtaaattcacTTACAACTTAATTCTGACAGCTTTTACTTCAGTG |
| ADH2p-SSA1p-F | cgaatatatacacacatatgtttgtttgaagagactaatcaaagaatcgttttc |
| ADH2p-R | tgtgtattacgatatagttaatagttgatagttgattgtatg |
| DPP1A-F | ACTATTATTAAGGCGCTTCTGTTTTTAGTCAAC |
| DPP1A-TPI1p-R | GTCCTCACAGTTTAAATTTTGGTCGTTTGCTATGATTTAATTCTGATTCTTTG |
| TPI1p-F | ATTTAAACTGTGAGGACCTTAATACATTCAGACAC |
| TPI1p-R | TTTTAGTTTATGTATGTGTTTTTTGTAGTTATAGATTTaagcaagaaaag |
| YlACL1-TPI1p-F | CACATACATAAACTAAAAATGTCCGCTAATGAAAACATCTCTAGATTCG |
| YlACL1-TTYS1t-R | GAAAACGGATTAAGCTATGCTTAAGATCTTGTTTTAGCTTTAGAAACGTCACCAG |
| TTYS1t-F | GCATAGCTTAATCCGTTTTCACGATTca |
| TTYS1t-R | ATTGGAATGTTCTATGTAATTTGTGTGACCTG |
| DPP1B-TTYS1t-F | CATAGAACATTCCAATaataaaaaagaatatatacTCCACATGACATACGAAATATACG |
| DPP1B-R | TTATACATAGTATGTGTTAAGGGGAACggaa |
| TEF1-DPP1A-F | CATAGCAAACGACCAAACCACACACCATAGCTTCAAAATGTTTCTAC |
| TEF1p-R | TTTGTAATTAAAACTTAGATTAGATTGCTATGCTTTCTTTCTAATG |
| YlACL2-TEF1p-F | CTAAGTTTTAATTACAAAATGTCCGCTAAATCTATCCATGAAGCT |
| YlACL2-ENO2t-R | ATTCTTAGTTAAAAGCACTTTAAACACCTAATGGTGTAGAAGCTTCTGTAG |
| ENO2t-F | AGTGCTTTTAACTAAGAATTATTAGTCTTTTCTGCTTATTTTTTC |
| ENO2t-DPP1B-R | gtatatattcttttttattAGGTATCATCTCCATCTCCCATATGCATATC |
| AnACLa-TPI1p-F | CACATACATAAACTAAAAATGTCCGCTAAATCTATCTTCGAAGCTG |
| AnACLa-TTYS1t-R | ACGGATTAAGCTATGCTTAAGCAGTACCAAACTCCTTGATATCAGTCTG |
| AnACLb-TEF1p-F | CTAAGTTTTAATTACAAAATGCCAGCTGCTCCATTAGTTTCa |
| AnACLb-ENO2t-R | CTTAGTTAAAAGCACTTTAAACATTAACTTCAACTCTACCTTCAGAACCAC |
| MmACL-TPI1p-F | CACATACATAAACTAAAAATGTCCGCTAAAGCTATTTCTGAAC |
| MmACL-TTYS1t-R | GAAAACGGATTAAGCTATGCTTACATAGACATATGTTCTGGCAAAACGTAG |
| X2A-F | cgtctatgaggagactgttagttggatatc |
| X2A-R | gaccacttcgagagcaagttgc |
| FBA1p-X2A-F | cttgctctcgaagtggtcTGGGTCATTACGTAAATAATGATAGGAATGGG |
| FBA1p-R | TTTGAATATGTATTACTTGGTTATGGTTATATAtgacaaaagaaaaag |
| ACC1-FBA1p-F | CAAGTAATACATATTCAAAATGAGCGAAGAAAGCTTATTCGAGTC |
| ACC1-S659A-R | AGCTAGTTGACGCAGTATGATATCAC |
| ACC1-S659A-F | CATACTGCGTCAACTAGCTGATGGTGGTCTTTTGATTGCC |
| ACC1-S1157A-R | AGCAACAGCCCTGTTCATACC |
| ACC1-S1157A-F | GTATGAACAGGGCTGTTGCTGTTTCAGATTTGTCATATGTTGCAAAC |
| ACC1-R | TTATTTCAAAGTCTTCAACAATTTTTCTTTATCATCGGTAG |
| TJID1t-ACC1-F | GTTGAAGACTTTGAAATAAAATCACCTATTGCGCCGCTC |
| TJID1t-X2B-R | tgagggccgattatgcaggAAAACGGAATCGATGTTTCaacttttcaatact |
| X2B-F | cctgcataatcggccctcac |
| X2B-R | ctcgccaaggcattaccatcc |
| PGK1p-X4A-F | gtcgccattcaagagcagGGGCCAGAAAAAGGAAGTGTTTCC |
| PGK1p-R | tgttttatatttgttGTAAAAAGTAGATAATTACTTCCTTGATGATC |
| YHM2-PGK1p-F | CaacaaatataaaacaATGCCATCTACCACTAATACTGCTG |
| YHM2-PGI1t-R | ATATTTAAGAGCGATTTGTCTAATGTTTGGCAACTGGGGTTTCAC |
| PGI1t-F | ACAAATCGCTCTTAAATATATACCTAAAGAACATTAAAGC |
| PGI1t-X4B-R | tcttcccatgcctgttGGTATACTGGAGGCTTCATGAGTTATGTC |
| X4B-PGI1t-F | GAAGCCTCCAGTATACCaacaggcatgggaagattcgc |
| TEF1p-X4A-F | gtcgccattcaagagcagCCACACACCATAGCTTCAAAATGTTTC |
| TEF1p-R | TTTGTAATTAAAACTTAGATTAGATTGCTATGCTTTCTTTCTAATG |
| PYC1-TEF1p-F | GTTTTAATTACAAAATGTCGCAAAGAAAATTCGCCG |
| PYC1-TPS1t-R | CATTTGCATCGGGTTCATCATGCCTTAGTTTCAACAGGAACTTG |
| TPS1t-F | TGAACCCGATGCAAATGAGACG |
| TPS1t-X4B-R | tcttcccatgcctgttttttgtcttcttcaaattgaaGTTACTTTCTAAAATGGC |
| TPI1p-X4A-F | gtcgccattcaagagcagATTTAAACTGTGAGGACCTTAATACATTCAGACAC |
| TPI1p-R | TTTTAGTTTATGTATGTGTTTTTTGTAGTTATAGATTTaagcaagaaaag |
| IDP2-F | CACATACATAAACTAAAAATGACAAAGATTAAGGTAGCTAACCCCATTG |
| IDP2-R | TTACAATGCAGCTGCCTCGAAC |
| TTYS1t-IDP2-F | GAGGCAGCTGCATTGTAAGCATAGCTTAATCCGTTTTCACGATTc |
| TTYS1t-X4B-R | gaatcttcccatgcctgttATTGGAATGTTCTATGTAATTTGTGTGACCTG |
| RPRS424-F | cagcttttgttccctttagtgaggg |
| RPRS424-R | cgcgcgctcactggccgtcg |
| PGK1p-pRS424-F | ctaaagggaacaaaagctgGGGCCAGAAAAAGGAAGTGTTTCC |
| PGK1p-R | tgttttatatttgttGTAAAAAGTAGATAATTACTTCCTTGATGATCtg |
| MpOMT-F | Caacaaatataaaacaatggttgctgatgaagaagttcgtg |
| MpOMT-R | CTCATTTGCATCGGGTTCAttacgggtacgcttcgataacga |
| TPS1t-F | TGAACCCGATGCAAATGAGACG |
| TPS1t-pRS424-R | cggccagtgagcgcgcgttttgtcttcttcaaattgaaGTTACTTTCTAAAATGGC |
| CrOMT6-F | CaacaaatataaaacaATGGATCTGCAGACCGCTG |
| CrOMT6-R | CTCATTTGCATCGGGTTCATTACGGGTAAACTTCGATGATAGAACGG |
| spnK-F | CaacaaatataaaacaATGAGCACCACCCACGAAATTG |
| spnk-R | CTCATTTGCATCGGGTTCATTATTCATCTTCAGCGCTGTTAACATCCG |
| SOMT2-F | CaacaaatataaaacaATGGCTTCTTCTTTGAACAACGGTAG |
| SOMT2-R | CTCATTTGCATCGGGTTCATTAGGGGTAGATTTCAATTAAAGACAAGTAACC |
| GeHI4'OMT-F | CaacaaatataaaacaATGGACTTCTCTTCTTCTAACGGTTCTG |
| GeHI4'OMT-R | CTCATTTGCATCGGGTTCATTATGGGAAGACTTCGATTAAAGACTTAAAACC |
| pY26-F | gagctccagcttttgttccctt |
| pY26-R | tcttccgcttcctcgctcact |
| ADH2p-pY26-F | gaacaaaagctggagctcTGTTTGTTTGAAGAGACTAATCAAAGAATCgttttc |
| ADH2p-AtGT-R | CAACCGGGGTGCCCATTGTGTATTACGATATAGTTAATAGTTGATAGTTGATTGTATGC |
| AtGT-F | ATGGGCACCCCGGTTG |
| AtGT-R | TCATAAGAAATTCGCTTAAACTTTTTCTTTCTGCAGTTTAACCAGG |
| ADH1t-F | GCGAATTTCTTATGATTTatgatttttattattaaataagtta |
| ADH1t-pY26-R | gagcgaggaagcggaagagcgCCGGTAGAGGTGTGGTCAATAAGAG |
| ADH6p-pY26-F | gaacaaaagctggagctcTACCTCACCTGAgttttgcttttttctcTG |
| ADH6p-R | CTTCAACCGGGGTGCCCATGATTTTGgcttttcttgttgttgtgttg |
| HXT7p-pY26-F | gaacaaaagctggagctcGGCTGCTACGggatgttttttttttac |
| HXT7p-R | CTTCAACCGGGGTGCCCATTTTTTgattaaaattaaaaaaactttttgtttttgtgt |
| EXG1A-F | GAGCCTGAGACAAGCCCGTAAC |
| EXG1A-R | TTTAGTTGGTAATTAActagaaaaagaaagtaaacaaaaaatcaaagg |
| EXG1B-EXG1A-F | gTTAATTACCAACTAAAGTCGCCCTCAGTCCGCT |
| EXG1B-R | gtTGTTTAAGTTCTTTTATCCTTCCTTAGATAACACCTTAAAG |
| XII2A-F | CGGCATGCAAACATCTACACAATTAG |
| XII2A-R | CATAACGCGTTACACGGAAGGAGAG |
| PGK1p-XII2-F | CTCCTTCCGTGTAACGCGTTATGGGGCCAGAAAAAGGAAGTGTTTCC |
| TPS1t-2-R | ttttgtcttcttcaaattgaaGTTACTTTCTAAAATGGCT |
| ADH2p-TPS1t-F | caatttgaagaagacaaaaTGTTTGTTTGAAGAGACTAATCAAAGAATCgttttc |
| ADH1t-XII2-F | GAGAGTCGCCGATAGTAGCCGGTAGAGGTGTGGTCAATAAGAG |
| XII2B-F | CTACTATCGGCGACTCTCTCGAAATTTTTC |
| XII2B-R | GAGCGAACGTAAGAGAGGTTAATGTC |
| SHM2p-F | ctagTTAATTACCAACTAAAtgtagaaaaaaaaaaagaaaaaagaagtgaaatttttc |
| SHM2p-R | GTTAAGGAGGATTCGGTTTAAGCTGTTATG |
| Cm1,2RhaT-F | ACCGAATCCTCCTTAACATGGATACTAAGCACCAAGATAAGCCATC |
| Cm1,2RhaT-R | ACAATGAGGTTCAAATTATTCAGACTTCTTAACTAATTGCAACAATTTTTCAAC |
| SDH1t-F | TTTGAACCTCATTGTATTTTACGGAAAAGAATATCATAC |
| SDH1t-EXG1B-R | GAGCGGACTGAGGGCGACGACAGCACCCTTGTACAGCAATTC |
| LPP1A-F | aaagggGCAGAAGCAAGATTCTC |
| LPP1A-R | TAACACTTACAGAGTCCTATCAGGAAAGAATAAAAG |
| LPP1B-F | CCTTGGTAGAATATGACGAGTTTCCTT |
| LPP1B-R | CCAATCATGGTTTCATGGTCACTGG |
| TDH1p-LPP1-F | GTCATATTCTACCAAGGATGTCCCACCAGCCAACAC |
| TDH1p-R | tttgttttgtgTGTAAATTTAGTGAAGTACTGTTTTT |
| OlRHM-F | TCACTAAATTTACAcacaaaacaaaATGGCTTCTCATACCCCAAAGAAC |
| OlRHM-R | TTAAACCTTCTTATTTGGTTCAAAAACATACTTAATCAAGGAG |
| FBA1t-F | CCAAATAAGAAGGTTTAAGTTAATTCAAATTAATTGATATAGTTTTTTAATGAGTATTG |
| FBA1t-LPP1-R | GGACTCTGTAAGTGTTAGCTATCAAAAACGATAGATCGATTAGGATGAC |
| Ty4A-F | TGTTGGAACGAGAGTAATTAATAGTGACATGAGTTG |
| Ty4A-PGK1p-R | CTTCCTTTTTCTGGCCCATAACATGTTCAACTAATAGGTCTTTAACACAGCTTC |
| PGK1p-F | GGGCCAGAAAAAGGAAGTGTTTCC |
| TPS1t-R | ttttgtcttcttcaaattgaaGTTACTTTCTAAAATGGCT |
| LEU2-F | caatttgaagaagacaaaaaactgtgggaatactcaggtatcgtaag |
| LEU2-R | ttaagcaaggattttcttaacttcttcggc |
| Ty4B-F | gaaaatccttgcttaaGGTAGGTACATATATGAGGAATATGAGTCGTC |
| Ty4B-R | TGTTGATAATTAGAGGTTAAAAATTAGTATTAATGAAGAAGTGAGTACT |
| HOA-F | CGCAAGTCCTGTTTCTATGCCT |
| HOA-R | TTGATTGCTGCTTATGAGGATATGGATTTAGAG |
| TEF1p-HOA-F | CTCATAAGCAGCAATCAACCACACACCATAGCTTCAAAATGTTTC |
| TEF1p-R | TTTGTAATTAAAACTTAGATTAGATTGCTATGCTTTCTTTCTAATG |
| SAM1-F | CTAAGTTTTAATTACAAAATGGCCGGTACATTTTTATTCACTTCTG |
| SAM1-R | CTCATTTGCATCGGGTTCATTAGAACTTCAAAGTCTTAGGCTTTTCCCATG |
| TPS1t-F | TGAACCCGATGCAAATGAGACG |
| PGK1p-TPS1t-F | caatttgaagaagacaaaaGGGCCAGAAAAAGGAAGTGTTTCC |
| MET6-F | ACaacaaatataaaacaATGGTTCAATCTGCTGTCTTAGGGT |
| MET6-R | TTAATTCTTGTATTGTTCACGGAAGTACTTGG |
| CYC1t-MET6-F | GAACAATACAAGAATTAAACAGGCCCCTTTTCCTTTGTC |
| CYC1t-HO-R | cttttattaCATACAACGCAAATTAAAGCCTTCGAGCGTCC |
| HOB-F | GTTGTATGtaataaaagtaaaatttaatattttggatgaaaaaaacC |
| HOB-R | TTCCAAGTCCAAGATTGAAGCTGCT |
| SAM2-F | CTAAGTTTTAATTACAAAATGTCCAAGAGCAAAACTTTCTTATTTACCTC |
| SAM2-R | CATTTGCATCGGGTTCATTAAAATTCCAATTTCTTTGGTTTTTCCCATGAGTAC |
| GAL1A-F | TGGAACTTTCAGTAATACGCTTAACTGCTC |
| GAL1A-R | ACGTTAAAGTATAGAGGTATATtaacaattttttgttgataCTTTTATG |
| CDC19p-GAL1-F | CCTCTATACTTTAACGTACAGATTGGGAGATTTTCATAGTAGAATTCAGC |
| CDC19p-R | TGTGATGATgttttatttgttttgattGGTGTCTTG |
| ADO1-GAL1,10-F | caaataaaacATCATCACAATGACCGCACCATTGGTAGTATTG |
| AOD1-R | CTATTTAGAGTAAGATATTTTTTCGGAAGGGTAAGAGG |
| DIT1t-ADO1-F | ATCTTACTCTAAATAGATAAAGTAAGAGCGCTACATTGGTCTACC |
| DIT1t-R | gtTGTTCTGAacaaagtaACTTGTTACTCCGCAACGCT |
| GAL1B-F | tactttgtTCAGAACAacttctcatttttttctactc |
| GAL1B-R | atttcttttcctccTCGCGCTTG |
| XII3A-F | TGTGCCCCTTAAAATTCATATACACTTTATGTTTAATTCG |
| XII3A-R | GAATGAGCAGGTACCCCTTATTATAATGATTAATACTTAC |
| GAL1.10-XII3-F | AGGGGTACCTGCTCATTCTTATAttgaattttcaaaaattcttactttttttttggatg |
| GAL1,10-R | TATAGTTTTTTCTCCTTGACGTTAAAGTATAGAGGTATATtaac |
| SAH1-GAL1,10p-F | CAAGGAGAAAAAACTATAATGTCTGCTCCAGCTCAAAACTAC |
| SAH1-R | TCAATATCTGTAGTGGTCGGCCTTG |
| PGI1t-F | GACCACTACAGATATTGAACAAATCGCTCTTAAATATATACCTAAAGAACATTAAAG |
| PGI1t-XII3B-R | CCTAATTAGCTCTATGCCTTTGAAACTAAATTTGTATATTGTTTGTCAGAAATAGGAC |
| XII3B-F | GCATAGAGCTAATTAGGTTTGAGTTAAGGAAAAAAATC |
| XII3B-R | GAACTTACAAGCTGATTTTGGTTCTTTTCCA |
| DIT1t-XII3A-R | AGGGGTACCTGCTCATTCACTTGTTACTCCGCAACGCT |
| DIT1t-F | ATCTTACTCTAAATAGATAAAGTAAGAGCGCTACATTGGTCTACC |
| XII4A-F | GTATCCGGCTGTTCCTTCATAGC |
| XII4A-R | GCCATAGTATGTGTGATGGAAAAAAACTATCG |
| FBA1t-F | GTTAATTCAAATTAATTGATATAGTTTTTTAATGAGTATTGAATCTG |
| FBA1t-XII4-R | CATCACACATACTATGGCGCTATCAAAAACGATAGATCGATTAGGATGAC |
| STR2-F | tgaaaattcaaTATAAATGATATCTAGAACCATTGGTGAATCTATTCC |
| STR2-R | CAATTAATTTGAATTAACTTATTCCCCTAAAGCTTTCTCAATGGCAC |
| GAL1.10p-F | TTTATAttgaattttcaaaaattcttactttttttttggatgGACG |
| GAL1,10p-R | TATAGTTTTTTCTCCTTGACGTTAAAGTATAGAGGTATATtaac |
| MET2-F | CAAGGAGAAAAAACTATAATGTCGCATACTTTAAAATCGAAAACGCTC |
| MET2-R | CTCATTTGCATCGGGTTCACTACCAGTTGGTAACTTCTTCGGCCT |
| XII4B-F | caatttgaagaagacaaaaATTCCCCATTAGAGTCAAATAAAAGGATGC |
| XII4B-R | TTTCTGCTGTACCTGGATGGTCATTTC |
| EGH1A-F | gacgaACCAGATTGTCAAATAAACATTGttaattg |
| EGH1A-TPI1p-R | GGTCCTCACAGTTTAAATATGATCTGTTCTTattaaattcaaacaaTTTAGGTacg |
| TPI1p-F | ATTTAAACTGTGAGGACCTTAATACATTCAGACAC |
| TPI1p-R | TTTTAGTTTATGTATGTGTTTTTTGTAGTTATAGATTTaagcaagaaaag |
| vHB-F | CACATACATAAACTAAAAATGTTGGACCAGCAGACTATCAAC |
| vHB-R | CTACTCAACAGCCTGGGCGT |
| PGI1t-vHB-F | ACGCCCAGGCTGTTGAGTAGACAAATCGCTCTTAAATATATACCTAAAGAACATTAAAG |
| PGI1t-EGH1-R | TGgttcaatttttgtCTTTGAAACTAAATTTGTATATTGTTTGTCAGAAATAGGAC |
| EGH1B-F | acaaaaattgaacCAAAAGCTATAGTagtcatatatatatatatacacc |
| EGH1B-R | CAAGCAAAGAGAAGTGGAATGATATAGATCAG |
| YPRCδ15CA-F | GTAACTCTACTCCGCCTGTGGTTTC |
| YPRCδ15CA-R | gaaattttaaaactACATTAATGTGttagtttttctttc |
| SHM2p-YPRCδ15C-F | GTagttttaaaatttctgtagaaaaaaaaaaagaaaaaagaagtgaaatttttc |
| SHM2p-PGM2-R | CAATTTGAAATGACATGTTAAGGAGGATTCGGTTTAAGCTGTTATG |
| PGM2-F | ATGTCATTTCAAATTGAAACGGTTCCCA |
| PGM2-R | cttgaggcttttgacattTTAAGTACGAACCGTTGGTTCTTCAGTTC |
| Ter22t-F | ataggttggcttccatgttggc |
| Ter22t-R | aatgtcaaaagcctcaaggtgcc |
| UGP1-ter22t-R | catggaagccaacctatTCAATGTTCCAAGATTTGCAAATTACCAGTAACG |
| UGP1-F | ATGTCCACTAAGAAGCACACCAAAAC |
| ARO7p-YPRCδ15C-R | GTGCTTCTTAGTGGACATATCTTATACCAATTTTATGCAGGATGCTGAGTG |
| ARO7p-YPRCδ15C-F | AGTTTGTTTGCGAAACGCTATATGCATCGAAGGGACTGC |
| YPRCδ15CB-F | GTTTCGCAAACAAACTTAAATATATGCtagaataaaaattaaC |
| YPRCδ15CB-R | GACAATTATCATATTATTCACCAATTAATCACAAGTTGGTAATG |
| SED1p-YPRCδ15C-F | GTagttttaaaatttcAATTATCTCCCAGACGGCACC |
| SED1p-PGM2-R | CAATTTGAAATGACATCTTAATAGAGCGAACgtattttattttgcttGTCTTTG |
| CYC1p-YPRCδ15C-F | TGTagttttaaaatttcaatttttttggaaaaccAAGAAATGAATTATATTTCCGTG |
| CYC1p-PGM2-R | CAATTTGAAATGACATTATTAATTTAGTGTGTGTATTTGTGTTTGTGTGTC |
| INO1p-YPRCδ15C-F | AGTTTGTTTGCGAAACagGCCGGTGCCGATG |
| INO1p-UGP1-R | GTGTGCTTCTTAGTGGACATTGttacttctttttcactggaaaaaaaaggga |
| ERG20p-YPRCδ15C-F | AGTTTGTTTGCGAAACGATCACGTAAAACAACAACCAGGAAAG |
| ERG20p-UGP1-R | GTGCTTCTTAGTGGACATTTCTACGTAATATTTTTGAGTTTATTGTGCCTATGTCAG |

**Table S2. Codon-optimized exogenous gene sequences involved in this study.**

| **Gene** | **Sequence (5’–3’)** |
| --- | --- |
| *SjCHS1* | *TTAACCTTGCAAAGGAACAGAATGTAAAACAACAGTTTCAACAGTCAAACCTGGACCAAAACCAAATAAAACACCCCATTCTAAACCTTCACCAGTTGTAGCTCTACCTTCTTCAACAGATTTTTTCCTCATTTCATCTAAAATAAACAAAACACATGCAGAACTCATATTACCATATTCAGATAAAACATGTCTAGTAGATCTTAATTTTTCTTCTTTTAAACCTAATTTAGCTTCAACTTGATCTAAAATAGCTGGACCACCTGGATGAGCGACCCAGAAAATAGAATTCCAATCAGAAATACCTACTGGAGCAAAAGCTTCAACTAAAGATTTTTCAATATTTTTAGAAATAATACCAGGAACATCTTTCAACAAATGGAAAGTCAAACCAACTTCTCTCAAATGACCATCAATTGCACCATCACTATCTGGTAAAATAGTTTGTGCAGCAGAAACTAATTGAAAAATTGGTCTTTCAACAGCAGTATCTGGATCAGCACCTATTATCATTGCAGCAGCACCATCTCCAAACAATGCTTGACCAACTAAAGAATCTAAGTGAGTATCAGATGGACCTCTAAAAGTAACAGCTGTTATTTCAGAACAAACTACTAAAACTCTAGCACCCTTATTATTTTCAGCTAGATCTTTAGCTAATCTTAAAACAGTACCACCAGCAAAACAACCTTGTTGATACATCATTAATCTTTTAACAGAAGGTCTTAAACCTAATAATTTAGTCAATTGGTAATCAGCACCTGGCATATCAACACCTGAAGTAGTACAAAACACCAAATGTGTAATTTTTGATTTAGGTTGACCCCATTCCTTAATTGCTTTACTAGCAGCTTCTTTACCTAATTTTGGAACTTCTACTACAACTATATCTTGTCTAACATCTAATGATGGAGCCATATATGCACACATATTAGGATTTTCTTTTAAAAATTCTTCAGTAACATGCATATATCTTTTCTTAATCATTGATTTTTCACACATTCTTTTGAATTTTTCTTTCAAATCAGTCATATGTTCAGAATCAGTAATTCTAAAATAATAATCAGGATAATCAGCTTGAGTAATACAATTAGAAGGTGTTGCAGTACCAAAAGCTAAAATTGTAGCTGGACCTTGAGATCTTTGAGCATTTCTAATTTCTTCAACTGTAACCAT* |
| *MsCHI* | *atggcagcaagcattacggcaatcacggttgaaaatctggaatatccggcggtcgttacctctccggtcacgggcaaatcatactttctgggcggtgccggtgaacgtggtctgaccattgagggtaactttatcaaattcacggcaattggcgtttatctggaagatatcgcggtcgcctcactggcggccaaatggaaaggtaaaagctctgaagaactgctggaaaccctggatttttaccgtgacattatctcaggcccgttcgaaaaactgatccgtggttcgaaaattcgcgaactgagcggcccggaatattctcgcaaagtcatggaaaactgcgtggctcatctgaaatccgtcggcacgtacggtgacgcagaagctgaagcgatgcagaaatttgccgaagcattcaaaccggtgaattttccgccgggtgccagtgttttctatcgtcaatccccggatggcatcctgggtctgtcattttcgccggacaccagcatcccggaaaaagaagcagctctgattgaaaataaagctgtgagttccgcggttctggaaacgatgattggcgaacacgcggtttctccggatctgaaacgctgtctggctgctcgcctgccggctctgctgaatgaaggtgcctttaaaatcggtaactga* |
| *Pc4CL* | *atgggtgactgcgttgccccgaaagaggatctgatcttccgcagcaaactgccggacatttacattccaaagcatctgccgctgcatacctattgttttgagaacatcagcaaggttggcgacaagagctgtctgatcaacggcgcaaccggcgaaacctttacctacagccaggttgagctgctgtcccgtaaagttgccagcggcctgaacaagctgggcattcaacaaggtgataccattatgctgctgctgccgaactccccggagtactttttcgctttcctgggtgcgagctatcgcggtgcaatcagcactatggcgaacccattctttaccagcgcagaagtgatcaagcaactgaaagcgagccaagcgaagctgattatcacccaggcatgctatgttgacaaggttaaggactacgcagcggagaaaaacatccagatcatttgtattgacgatgcaccgcaggattgcctgcactttagcaagctgatggaagcggatgagagcgaaatgccggaagtggttattaacagcgatgatgtggtggcactgccgtacagctctggcaccaccggcctgccgaaaggcgttatgctgacccacaagggtctggttaccagcgttgcacaacaggtggatggtgataacccgaacctgtatatgcactccgaggatgttatgatctgcatcctgccactgttccatatctatagcctgaacgctgttctgtgttgtggtctgcgtgcgggcgttaccattctgatcatgcaaaagttcgacattgtgccgtttctggagctgattcagaagtataaggttaccattggtccgtttgttccgccgatcgtgctggccatcgcgaaaagcccggttgttgacaagtacgacctgtctagcgtgcgcaccgttatgagcggtgcagcgccgctgggtaaagagctggaggacgctgttcgtgcgaaattcccgaacgcgaagctgggtcaaggctatggcatgaccgaagccggtccggttctggcgatgtgtctggcgttcgccaaagagccgtatgagattaagtctggcgcatgcggtaccgttgtgcgtaacgccgagatgaaaatcgttgacccagaaaccaacgcgtctctgccgcgtaaccagcgtggtgagatttgcatccgtggtgatcagattatgaaaggttacctgaacgacccggaaagcacccgcaccaccatcgacgaagagggttggctgcacaccggtgacattggtttcatcgacgatgacgatgaactgttcattgttgatcgtctgaaagaaatcattaagtacaaaggttttcaagttgctccggcggagctggaagcactgctgctgacccacccgaccatcagcgatgccgcggtggttccgatgattgacgagaaagcgggtgaagtgccagtggcgtttgttgtgcgtaccaacggttttaccaccaccgaagaagaaatcaaacaatttgtgagcaaacaggttgtgttctacaaacgtatcttccgcgttttcttcgttgacgctattccgaaatccccgagcggcaagattctgcgtaaggatctgcgcgctcgtattgcgagcggcgacctgccgaagtaa* |
| *YlACL1* | *ATGTCCGCTAATGAAAACATCTCTAGATTCGATGCTCCAGTTGGTAAAGAACATCCAGCTTATGAATTGTTCCACAATCATACAAGGTCCTTTGTTTACGGTTTGCAACCAAGAGCTTGTCAAGGTATGTTGGATTTTGATTTCATCTGCAAGAGGGAAAACCCATCTGTTGCTGGTGTTATATATCCATTTGGTGGTCAATTCGTCACTAAAATGTACTGGGGTACTAAAGAAACCTTGTTGCCAGTTTATCAGCAAGTTGAAAAGGCTGCTGCTAAACATCCAGAAGTTGATGTTGTTGTTAACTTCGCTTCTTCTAGATCTGTTTACTCTTCTACAATGGAGTTGTTGGAATACCCACAATTTCGTACTATCGCTATTATCGCTGAAGGTGTTCCAGAAAGAAGAGCTAGAGAAATTTTGCATAAGGCTCAAAAGAAGGGTGTTACAATTATCGGTCCAGCTACTGTTGGTGGTATTAAGCCAGGTTGTTTTAAAGTTGGTAACACAGGTGGTATGATGGATAATATTGTCGCTTCTAAGCTGTACAGACCAGGTTCTGTTGCTTATGTTTCTAAATCTGGTGGTATGTCTAACGAATTGAACAATATCATCTCCCACACTACAGATGGTGTTTATGAAGGTATTGCTATCGGTGGTGACAGATATCCAGGTACAACATTCATTGATCACATCTTGAGATACGAGGCTGATCCAAAATGTAAAATCATCGTTCTGCTGGGTGAAGTTGGTGGTGTTGAAGAATATAGAGTTATCGAAGCTGTCAAGAACGGTCAAATTAAGAAACCAATCGTCGCTTGGGCTATTGGTACTTGTGCTTCTATGTTTAAGACCGAAGTTCAATTCGGTCATGCTGGTTCTATGGCTAATTCTGATTTGGAAACTGCTAAGGCTAAGAATGCTGCTATGAAATCTGCTGGTTTTTACGTTCCAGATACATTTGAAGACATGCCAGAAGTTTTGGCTGAATTATATGAGAAGATGGTCGCTAAAGGTGAATTATCTAGAATCTCTGAGCCAGAAGTTCCAAAAATTCCAATTGATTACTCCTGGGCTCAAGAATTGGGTTTAATTAGAAAGCCAGCTGCTTTTATCTCCACAATTTCTGATGATAGGGGTCAAGAATTGTTGTATGCTGGTATGCCAATTTCTGAAGTTTTCAAAGAGGACATCGGTATTGGTGGTGTTATGTCTTTGTTATGGTTCAGAAGAAGGTTGCCAGATTATGCTTCTAAATTCTTGGAGATGGTCTTGATGTTGACTGCTGATCATGGTCCAGCTGTTTCTGGTGCTATGAATACTATTATCACCACTAGAGCTGGTAAAGATTTGATTTCTTCCTTGGTTGCTGGTTTGTTGACAATTGGTACAAGATTTGGTGGTGCTTTAGATGGTGCTGCTACTGAATTCACTACAGCTTATGATAAGGGTTTGTCTCCAAGACAATTCGTTGATACTATGAGAAAGCAGAACAAGTTGATCCCAGGTATTGGTCATAGAGTTAAATCTAGAAACAACCCAGACTTCAGAGTTGAATTAGTTAAGGATTTCGTCAAGAAGAACTTCCCATCTACTCAATTACTGGATTACGCTTTGGCTGTTGAAGAAGTTACAACATCTAAGAAGGACAACTTGATCTTGAACGTTGATGGTGCTATTGCTGTTTCTTTTGTTGATTTGATGAGGTCTTGCGGTGCTTTTACTGTTGAAGAAACAGAAGATTACCTGAAGAACGGTGTTTTAAACGGTTTGTTTGTCTTGGGTAGATCTATTGGTTTGATCGCTCATCATTTGGATCAAAAAAGGTTGAAGACCGGTTTATACAGACATCCATGGGATGATATTACCTATTTGGTTGGTCAAGAGGCTATTCAAAAGAAAAGAGTTGAGATCTCCGCTGGTGACGTTTCTAAAGCTAAAACAAGATCTTAA* |
| *YlACL2* | *ATGTCCGCTAAATCTATCCATGAAGCTGATGGTAAAGCTTTATTGGCTCATTTTCTGTCTAAGGCTCCAGTTTGGGCTGAACAACAACCAATTAATACTTTCGAGATGGGTACTCCAAAGTTGGCTTCTTTGACTTTTGAAGATGGTGTTGCTCCAGAACAAATTTTTGCTGCTGCTGAAAAAACCTACCCATGGTTGTTAGAATCTGGTGCTAAATTTGTCGCTAAGCCAGATCAATTAATCAAGAGAAGAGGTAAAGCCGGTTTGTTAGTTTTAAACAAGTCCTGGGAAGAGTGTAAACCATGGATTGCTGAAAGAGCTGCTAAACCAATTAACGTTGAAGGTATTGACGGTGTTTTGAGAACTTTTCTAGTCGAACCATTCGTTCCACATGATCAAAAACATGAATACTACATCAACATCCACTCCGTTAGAGAAGGTGACTGGATTTTATTTTACCACGAAGGTGGTGTTGACGTTGGTGACGTTGATGCTAAAGCTGCTAAAATTTTGATCCCAGTTGACATTGAGAACGAATATCCATCTAACGCTACTTTGACTAAGGAATTGTTGGCTCATGTTCCAGAAGATCAACATCAAACTTTGTTGGATTTCATCAACAGGTTGTACGCTGTTTATGTTGATTTGCAATTCACCTACCTGGAAATTAACCCATTGGTTGTTATCCCAACTGCTCAAGGTGTTGAAGTTCATTATTTGGATCTGGCTGGTAAATTGGATCAAACAGCTGAATTTGAGTGCGGTCCAAAATGGGCTGCTGCTAGATCTCCAGCTGCTTTAGGTCAAGTTGTTACAATTGATGCTGGTTCTACTAAGGTTTCTATCGATGCTGGTCCAGCTATGGTTTTTCCAGCTCCATTTGGTAGAGAATTATCTAAAGAGGAGGCTTATATCGCTGAATTGGATTCTAAAACCGGTGCTTCTTTGAAATTGACTGTTTTAAACGCCAAGGGTAGAATTTGGACTTTAGTTGCTGGTGGTGGTGCTTCTGTTGTTTATGCTGATGCTATTGCTTCTGCTGGTTTTGCTGATGAATTGGCTAATTATGGTGAGTATTCCGGTGCTCCAAATGAAACTCAAACTTATGAATACGCCAAGACAGTTTTGGATTTGATGACAAGAGGTGACGCTCATCCAGAAGGTAAAGTTTTATTCATTGGCGGTGGTATTGCTAATTTCACACAAGTTGGTTCTACCTTTAAGGGTATTATCAGAGCTTTCAGAGACTATCAATCCTCTTTACATAACCACAAGGTCAAAATCTACGTCAGAAGAGGTGGTCCAAATTGGCAAGAAGGTTTGAGATTAATCAAGTCTGCTGGTGACGAATTGAATTTGCCAATGGAAATATATGGTCCCGATATGCATGTTTCTGGTATTGTTCCATTGGCTTTATTGGGTAAAAGGCCAAAAAACGTTAAGCCATTTGGTACAGGTCCATCTACAGAAGCTTCTACACCATTAGGTGTTTAA* |
| *AnACLa* | *ATGTCCGCTAAATCTATCTTCGAAGCTGATGGTAAAGCTATTTTGAACTACCATCTGACTAGGGCTCCAGTTATTAAGCCAACACCATTACCACCATCTAATACTCATAACCCACCACCAAAATTGGCTTCTTTATATTTCCCAGACGACTTGTCTGTTAAGGATGTTTTAGATCAGGCTGAAGTTACTTACCCATGGTTGTTAACTCCAGGTTCTAAATTTGTCGCTAAGCCAGATCAATTGATCAAAAGAAGAGGTAAATCCGGTTTGTTGGCTTTAAATAAGACCTGGGCTGAAGCTAGAGAATGGATTGAAGCTAGAGCTACAAAAGAACAACAGGTTGAAACTGTTGTCGGTGTTTTAAGACATTTCTTGGTTGAACCATTCGTCCCACATCCACAAGAAACTGAATATTATATCAACATCCACTCCGTCAGAGAAGGTGACTGGATTTTATTCACTCATGAAGGTGGTGTTGACGTTGGTGACGTTGATGCTAAAGCTGAAAAATTACTGATCCCAGTTAACTTGAAGAACTACCCATCTAATGAGGAAATCGCTTCTGCTTTATTGTCTAAAGTCCCAAAAGGTATCCACAATGTTTTGGTTGATTTCATCTCCAGACTGTATGCTGTTTATGTTGATTGTCAGTTCACCTATCTGGAAATTAACCCATTAGTCGTTATCCCAAACGCTGATGCTACTTCTGCTGATGTTCATTTTCTAGATCTGGCTGCTAAATTGGATCAAACTGCTGAATTTGAGTGCGGTACTAAATGGGCTGTTGCTAGATCTCCAGCTAATTTGGGTTTAGCTGCTTTACCAACTTCTGATAAAGTCAATATCGACGCTGGTCCACCAATGGAATTTCCAGCTCCATTTGGTAGAGAATTGTCTAAAGAAGAGAAGTTCATCTCCGATATGGATGCTAAAACAGGTGCTTCTTTGAAATTGACTGTCTTAAACCCAAACGGTAGAGTTTGGACATTGGTTGCTGGTGGTGGTGCTTCTGTTGTTTATGCTGATGCTATTGCTTCTGCTGGTTTTGTTTCTGAATTGGCTAATTACGGTGAGTATTCTGGTGCTCCAACTGAAACTCAAACTTTTAATTACGCCAGGACAATCTTGGATTTGATGTTAAGATCCCCAATCCATCCAGATGGTAAAGTTTTGTTTATCGGTGGTGGTATTGCTAATTTCACTAATGTTGCTTCCACCTTTAAGGGTGTTATTAGAGCTTTGAGAGAGGTTGCTCCAGTTTTGAATGAACATAAAGTCCAAATCTGGGTCAGAAGAGCTGGTCCAAATTATCAAGAAGGTTTGAAAAACATCAAGGCCGTTGGTGAAGAATTGGGTTTGAATATGCATGTTTACGGTCCAGAAATGCATGTTTCTGGTATTGTTCCATTGGCTTTACAAGGTAAACAGACTGATATCAAGGAGTTTGGTACTGCTTAA* |
| *AnACLb* | *atgcctgccgctcctcttgtcagcactgccaacggccctaacgccaacgataacatcactcgcttcgagcctcccagccgagtgcgctctcccttcgccgatgccctcttccacaacaagacaagatgtttcgtatacggtatgcagccccgggctgtccagggtatgctggatttcgacttcatctgcaagcgttccactccttccgttgccggtatcatctacacattcggcggtcaattcgtcagcaagatgtactggggtaccagtgaaaccctcctccctgtttaccaggacaccgcaaaggccatggccaagcaccccgacgttgacaccgttgtcaacttcgcctcttcccgttccgtctacagctctactatggagctgatgcagtaccctcagatcaaatgcattgccatcattgcagagggtgttccagaaaggcgagctcgtgaaatccttgtcaccgctaaggagaagggcatcaccatcattggacctgctacagtcggtggtatcaagcctggcgctttcaaaattggtaacactggtggtatgatggacaacattgtcgcttccaagctctaccgcaagggatccgttggttatgtgtccaagtctggtggaatgtccaacgaattgaacaacatcatctcccaaactactgacggtgtttacgagggtgttgctattggaggtgaccgttaccccggtactactttcatcgaccacctccttcgttaccaagccgagcctgagtgcaagatccttgttctgctcggtgaggttggtggtgttgaggaataccgtgtcattgaggctgtcaagaacggtgtgatcaccaaacccatcgtcgcctgggccatcggtacttgcgctagcatgttcaagactgaggtccagtttggtcacgctggtgcctctgccaactccgacctggagactgctgttgctaagaacaaggctatgagagaagccggtatctacgtccctgacacattcgaggacatgcccgccgtcctcaagaaggtctacgaggagcaggttcagaacggtgttatcaagcctcagcctgagcctgttccccctaagattcccattgactactcttgggcccaggagctcggtcttattcgtaagcctgctgctttcatctccaccatctccgacgaccgtggccaggagctcttatatgccggcatgcccatctctgatgtcttcaaggaggacattggtatcggaggtgtcatgtccttgctctggttccgccgccgcctgcccagctacgctaccaagttcttggagatggttctcatgctcacagctgaccacggtcccgctgtgtctggcgccatgaacactatcatcacaactcgtgccggcaaggacctcatcagtgcccttgtctctggtcttctcaccattggatcccgctttggtggtgccctagatggcgctgccgaggagttcaccaaggctttcgacaagggcatgagccctcgtgacttcgttgacaccatgagaaaggagaacaagctgattcctggaattggccaccgtatcaagtcccgcaacaaccccgatctgcgtgttgagctggttaaggaatacgtcaagaagcacttccccagcaccaagcttctggattacgccattgctgtcgagactgtcaccacatccaagaaggacaacctgattcttaacgtcgacggttgcatcgctgtttgcttcgtcgatctcatgcgcaactgcggtgctttctccgccgaggaatccgaggactacatgaagatgggtgtcctcaacggtcttttcgttctaggccgtagcatcggtctgattgcccactaccttgatcagaagagactgcgcactggtctttaccgccacccttgggatgacatcacgtacctgctccccgccctgcaaaagggtggctcggagggtcgtgttgaggtcaacgtataa* |
| *MmACL* | *ATGTCCGCTAAAGCTATTTCTGAACAAACTGGTAAAGAGTTGCTGTATAAGTACATCTGTACCACTTCTGCTATCCAAAATAGATTCAAGTACGCTAGAGTCACACCAGATACTGATTGGGCTCATTTGTTGCAAGATCATCCATGGTTATTGTCTCAATCTTTGGTTGTTAAGCCCGATCAATTGATTAAGAGAAGAGGTAAACTGGGTTTGGTTGGTGTTAATTTGTCTTTGGATGGTGTTAAGTCCTGGTTGAAACCAAGATTGGGTCATGAAGCTACAGTTGGTAAAGCTAAAGGTTTTCTAAAGAACTTCCTGATCGAGCCATTTGTTCCACATTCTCAAGCTGAAGAATTCTATGTTTGCATCTACGCTACTAGAGAAGGTGACTATGTTTTATTCCACCATGAAGGTGGTGTTGATGTTGGTGACGTTGATGCTAAAGCTCAAAAATTGTTGGTCGGTGTTGATGAAAAGTTGAATACAGAAGACATCAAGAGGCATTTGTTGGTTCATGCTCCAGAAGATAAAAAGGAAGTTTTGGCTTCTTTCATCTCCGGTTTATTCAATTTCTACGAGGATTTGTACTTCACCTACTTAGAAATCAACCCATTGGTTGTTACCAAGGATGGTGTTTATATCTTGGATTTGGCTGCTAAAGTCGATGCTACAGCTGATTATATTTGCAAAGTCAAGTGGGGTGACATTGAATTTCCACCACCATTTGGTAGAGAAGCTTATCCAGAAGAAGCTTATATCGCTGATTTGGATGCTAAATCTGGTGCTTCTTTGAAATTGACACTGTTGAATCCAAAGGGTAGAATTTGGACTATGGTTGCTGGTGGTGGTGCTTCTGTTGTTTATTCTGATACAATCTGCGACTTGGGTGGTGTTAATGAATTAGCTAATTACGGTGAGTACTCCGGTGCTCCATCTGAACAACAAACTTATGATTACGCTAAGACCATCTTGTCTTTGATGACAAGAGAAAAGCACCCAGAAGGTAAAATTTTGATCATCGGTGGTTCTATCGCTAATTTTACTAACGTTGCTGCTACTTTCAAGGGTATTGTTAGAGCTATTAGGGATTACCAAGGTCCATTGAAAGAACATGAAGTTACAATCTTCGTCAGGAGAGGTGGTCCAAATTATCAAGAAGGTTTAAGAGTCATGGGCGAAGTTGGTAAAACAACTGGTATTCCAATCCATGTTTTCGGTACTGAAACACATATGACTGCTATTGTTGGTATGGCTTTAGGTCATAGACCAATTCCAAATCAACCACCAACTGCTGCTCATACTGCTAATTTTCTATTGAACGCTTCCGGTTCTACATCTACTCCAGCTCCATCTAGAACTGCTTCTTTTTCTGAATCTAGGGCTGATGAAGTTGCTCCAGCTAAAAAAGCTAAACCAGCTATGCCACAAGATTCTGTTCCATCTCCAAGATCTTTGCAAGGTAAATCTGCTACTTTGTTCTCTAGACATACCAAAGCTATCGTTTGGGGTATGCAAACAAGAGCTGTTCAAGGTATGTTGGATTTTGATTACGTCTGTTCTAGGGATGAACCATCTGTTGCTGCTATGGTTTATCCATTCACTGGTGACCATAAACAAAAGTTTTACTGGGGTCATAAGGAGATTTTGATCCCAGTTTTTAAGAACATGGCCGATGCTATGAAAAAGCATCCAGAAGTTGATGTTCTGATCAATTTCGCTTCTTTGAGATCTGCTTACGATTCTACTATGGAAACTATGAACTACGCTCAAATCAGAACTATCGCTATTATCGCTGAAGGTATTCCAGAAGCTTTAACAAGAAAGTTGATCAAGAAGGCTGACCAAAAAGGTGTTACTATTATCGGTCCAGCTACAGTTGGAGGTATTAAGCCAGGTTGTTTTAAAATCGGTAACACTGGTGGTATGTTGGACAATATTTTGGCTTCTAAGCTGTACAGACCAGGTTCTGTTGCTTATGTTTCTAGATCTGGTGGTATGTCTAACGAATTGAATAACATCATCTCCAGGACTACTGATGGTGTTTACGAAGGTGTTGCTATTGGTGGTGACAGATATCCAGGTTCTACTTTTATGGATCACGTTTTAAGGTACCAGGATACTCCAGGTGTTAAAATGATTGTTGTCTTGGGTGAAATCGGTGGTACTGAAGAATATAAAATCTGCAGAGGTATCAAGGAGGGTAGATTGACAAAACCAGTTGTTTGTTGGTGTATCGGTACTTGTGCTACTATGTTTTCTTCTGAAGTCCAATTCGGTCATGCTGGTGCTTGTGCTAATCAAGCTTCTGAAACTGCTGTTGCTAAAAATCAAGCTTTGAAGGAAGCTGGTGTTTTTGTTCCAAGATCTTTTGATGAGCTGGGTGAAATTATCCAATCTGTTTATGAGGACCTGGTTGCTAAAGGTGCTATTGTTCCAGCTCAAGAAGTTCCACCACCAACTGTTCCAATGGATTATTCTTGGGCTAGAGAATTGGGTTTGATTAGAAAACCAGCTTCTTTCATGACCTCTATTTGTGATGAAAGGGGTCAAGAATTGATCTATGCTGGTATGCCAATTACAGAAGTTTTCAAAGAGGAGATGGGTATTGGTGGTGTTTTGGGTTTATTATGGTTCCAAAGAAGGTTGCCAAAGTATTCTTGTCAATTCATCGAGATGTGCTTGATGGTTACAGCTGATCATGGTCCAGCTGTTTCTGGTGCTCATAATACTATTATCTGCGCTAGAGCTGGTAAAGATTTGGTTTCTTCTTTGACTTCCGGTTTATTGACTATCGGTGACAGATTTGGTGGTGCTTTAGATGCTGCTGCTAAAATGTTTTCTAAGGCTTTTGACTCCGGTATTATCCCAATGGAATTTGTTAACAAGATGAAGAAGGAGGGTAAATTGATCATGGGTATTGGACATAGAGTCAAATCTATCAACAACCCAGATATGAGAGTCCAAATTTTGAAGGATTTCGTCAAGCAACACTTCCCAGCTACTCCATTATTAGATTATGCTTTGGAGGTCGAAAAGATCACAACTTCTAAAAAGCCAAACCTGATCTTGAACGTTGATGGTTTTATCGGTGTTGCTTTTGTTGATATGCTGAGAAATTGCGGTTCTTTTACAAGAGAAGAGGCTGATGAATACGTTGATATTGGTGCTTTAAACGGTATCTTCGTTTTGGGTAGATCTATGGGTTTTATCGGACATTATCTGGATCAAAAGAGGTTAAAGCAGGGTTTGTATAGACATCCATGGGATGATATTTCCTACGTTTTGCCAGAACATATGTCTATGTAA* |
| *MpOMT* | *atggttgctgatgaagaagttcgtgttcgtgcggaagcatggaacaacgcgttcggttacatcaaaccgaccgcagttgcgaccgcggttgaactgggtctgccggatatcctggaaaaccacgatggtccgatgagcctgctggaactgagcgcggctaccgattgcccggccgaaccgctgcaccgtctgatgcgtttcctggttttccacggtatcttcaaaaagaccgcgaaaccgccgctgtctaacgaagcggtttactacgcgcgtaccgcgctgagccgcctgttcacccgtgacgaactgggtgacttcatgctgctgcagaccggtccgctgtctcagcacccggctggcctgaccgcgtccagcctgcgcaccggtaaaccgcagttcatccgtagcgtgaacggcgaagattcttggaccgatccggttaacggttaccacatgaaagttttctccgatgcgatggcggcgcacgcacgcgaaaccaccgcggcgatcgttcgttactgcccggcggcgttcgaaggtatcggtaccgttgttgatgttggtggccgtcacggcgttgcgctggaaaaactggttgcggcattcccgtgggtgcgtggtatctctttcgatctgccggaaatcgttgcgaaagcgccgccgcgcccaggcatcgaattcgttggtggttctttcttcgaatctgtaccgaaaggtgatctggttctgctgatgtggatcttgcacgattggtccgatgaaagctgcatcgaaatcatgaaaaaatgcaaagaagcgatcccgaccagcggtaaagttatgatcgtggatgcgatcgttgatgaagatggtgaaggtgatgatttcgcgggcgcgcgtctgagcctggatctgatcatgatggcggttctggcgcgtggtaaagaacgtacctaccgtgaatgggaatacctgctgcgtgaagcgggtttcaccaaattcgttgttaaaaacatcaacaccgttgaattcgttatcgaagcgtacccgtaa* |
| *CrOMT6* | *ATGGATCTGCAGACCGCTGAATTCCGTGAAGCGCAGGCTAAAATCTGGTCTCAGGCGTTCTCCTTCGCGAACTGCGCAGCGCTGAAATGCGCGGTTAAACTGGGTATTGCTGACGCGATCGATAACCACGATAAAAAAGCTCTGACCCTGTCTGAACTGACCGAAGAACTGTCTATCAAACCGTCTAAATCTCCGTTCCTGCAGCGTCTGATGCGTCAGCTGGTTAACGCTGGTTTCTTCACCGAAGCTAAACAGCTGCGTGATGATAATAAAGACGGCCGTACCACCACCGCGTACGCGCTGACCCCGGTTTCTCGCCTGCTGCTGAAAAACGAACAGTGGAACCTGCGCGGTATCGTTCTGACCATGCTGGACCCGGCGGAACTGAAAGCGTGGAGCGTTCTGAACGATTGGTTCAAAAACGATGATCCGACCGCGTTCCAGACCGCGCACGAAAAGAACTACTGGGATTACACCGCTGAAAACACCCAGCACTGCCAGATCTTTGAAGATGCAATGGCAAACGATTCTGTTCTGGTTAGCAAACTGCTGGTTACCGAATACAAATTCCTGTTCGAAGGCCTGACCAGCCTGATCGATCTGGGTGGTTCTACCGGTACCATCGCGAAAGCGCTGGCGAAGAGCTTCCCGAACCTGAAATGCACCGTTTTCGATCTGCCGCATGTGGTTGCGAACCTGGAAAGCACCAAAAACCTGGAATTCGTGGGTGGCGATATGTTCGAAAAACTGCCGCCGAGCAACGCGATCCTGCTGAAATGGATTCTGCACGATTGGAACGATGAAGATTGCGTGAAAATCCTGAAAAACTGCAAAAAAGCGATCCAGGAAAAAGGTAACGGTGGCAAAGTTATCATCATCGATACCGTTGTTTACAGCCAGAAAAACGAAAAAGAACTGGTTGATCTGCAGATCAGCATGGATATGGCGATGGTTATTAACTTCGCGGCTAAAGAACGTACCGAAGAAGAATGGGAACACCTGATCCGTGAAGCGGGCTTCTCTGGTCACAAAATCTTCCCGATGTATGATTTCCGTTCTATCATCGAAGTTTACCCGTAA* |
| *spnK* | *ATGAGCACCACCCACGAAATTGAAACCGTTGAACGTATCATCCTGGCGGCAGGTAGCTCTGCGGCTTCCCTGGCAGACCTGACCACCGAACTGGGCCTGGCGCGTATCGCGCCGGTTCTGATTGATGAAATTCTGTTCCGCGCTGAACCGGCGCCGGATATCGAACGTACCGAAGTTGCGGTGCAGATCACCCACCGTGGCGAAACCGTGGACTTCGTTCTGACCCTGCAGAGCGGTGAACTGATCAAAGCGGAACAGCGTCCGGTTGGCGATGTTCCGCTGCGTATCGGCTACGAACTGACCGATCTGATCGCGGAACTGTTTGGTCCGGGCGCGCCGCGTGCTGTTGGCGCTCGCAGCACCAACTTCCTGCGCACCACCACCTCTGGCTCTATCCCTGGTCCAAGCGAACTGAGCGATGGCTTCCAGGCGATTTCCGCGGTTGTGGCGGGTTGCGGCCACCGTCGTCCGGATCTGAACCTGCTGGCGTCCCATTACCGCACCGATAAATGGGGTGGCCTGCACTGGTTCACCCCGCTGTACGAACGCCACCTGGGTGAATTCCGTGACCGTCCGGTTCGTATCCTGGAAATCGGTGTTGGCGGCTACAACTTCGATGGTGGCGGCGGCGAATCTCTGAAAATGTGGAAACGCTACTTCCACCGTGGCCTGGTTTTTGGCATGGATGTTTTCGATAAGTCTTTCCTGGATCAGCAGCGTCTGTGCACTGTACGTGCGGATCAGAGCAAACCGGAAGAACTGGCTGCTGTGGATGATAAATACGGTCCGTTCGATATCATCATTGATGATGGCTCCCATATCAACGGCCACGTTCGTACCAGCCTGGAAACCCTGTTCCCGCGTCTGCGTAGCGGCGGTGTGTACGTGATCGAAGATCTGTGGACCACCTACGCACCGGGTTTCGGTGGCCAGGCACAGTGCCCGGCGGCACCGGGTACCACCGTTAGCCTGCTGAAAAACCTGCTGGAAGGCGTTCAGCACGAAGAACAGCCGCACGCGGGCAGCTACGAACCGAGCTACCTGGAACGCAACCTGGTGGGCCTGCACACCTATCACAACATCGCGTTCCTGGAAAAAGGTGTTAACGCGGAAGGTGGCGTTCCGGCGTGGGTTCCGCGTAGCCTGGATGATATCCTGCACCTGGCGGATGTTAACAGCGCTGAAGATGAATAA* |
| *SOMT2* | *ATGGCTTCTTCTTTGAACAACGGTAGAAAAGCTTCTGAAATCTTCCAAGGTCAAGCTTTATTGTACAAGCATTTGTTGGGTTTCATCGATTCTAAGTGTTTGAAGTGGATGGTTGAATTGGATATCCCAGATATTATCCACTCTCATTCTCATGGTCAACCAATTACTTTCTCTGAATTGGTTTCCATCCTGCAAGTTCCACCAACAAAAACTAGACAAGTTCAATCTCTGATGAGGTATTTGGCTCATAATGGTTTCTTTGAGATCGTTAGAATCCACGATAACATCGAAGCTTATGCTTTAACAGCTGCTTCTGAATTGTTGGTTAAGTCTTCTGAACTGTCTTTGGCTCCAATGGTTGAATATTTTCTGGAACCAAACTGCCAAGGTGCTTGGAATCAATTGAAAAGATGGGTTCATGAGGAGGATTTGACTGTTTTTGAAGTTTCTCTGGGTACACCATTTTGGGATTTTATTAACAAGGACCCAGCTTATAACAAGTCTTTTAACGAAGCTATGGCCTGTGATTCTCAAATGTTGAATTTGGCTTTCAGGGATTGTAACTGGGTTTTTGAAGGTTTAGAGTCTATCGTTGACGTTGGTGGTGGTACAGGTATTACTGCTAAAATTATCTGCGAGGCTTTCCCAAAATTGAAATGTATGGTTCTGGAGAGACCAAATGTTGTTGAAAATTTGTCCGGTTCTAACAACTTGACATTCGTTGGTGGTGACATGTTTAAATGTATCCCAAAAGCTGACGCTGTTTTGTTGAAATTGGTTTTGCATAACTGGAACGACAACGATTGTATGAAAATCTTGGAGAACTGCAAGGAAGCTATTTCTGGTGAATCTAAAACCGGTAAAGTCGTTGTTATCGATACTGTTATCAACGAAAACAAGGACGAAAGACAAGTTACAGAATTGAAGTTGCTGATGGATGTTCATATGGCTTGTATTATCAACGGTAAAGAGAGAAAGGAGGAAGATTGGAAAAAGTTATTCATGGAGGCTGGTTTTCAGTCTTATAAAATCTCTCCATTCACCGGTTACTTGTCTTTAATTGAAATCTACCCCTAA* |
| *GeHI4'OMT* | *ATGGACTTCTCTTCTTCTAACGGTTCTGAAGATACAGAATTGTCTCAAGCTCAAATCCATTTGTACAAGCATGTTTACAACTTCGTCTCTTCTATGGCTTTGAAATCTGCTATGGAATTGGGTATTGCTGATGTTATTCACTCTCATGGTAAACCAATCACTTTGCCAGAATTAGCTACTGCTTTAAACTTGAGACCATCTAAAATCGGTGTCTTACATAGATTCCTGAGATTATTGACCCACAATGGTTTCTTTGCTAAGACTACAGTTTCCAGAGGTGAAGGTGCTGAAGAAGAAACAGCTTATGGTTTGACACCACCATCTAAATTACTGGTTAAGTCTAACTCCACCTGTTTGGCTCCAATTGTTAAAGGTGCTTTGCATCCATCTTCTTTAGATATGTGGAGATCTTCTAAGAAGTGGTTTTTGGAAGACAACGAAGAATTGACTCTGTTTGAATCTGCTACAGGTGAATCTTTTTGGGAATTTTTGAACAAGGAGACCGAATCTGATACTTTATCTATGTTCCAGGAGGCTATGGCTGCTGATTCTCATATGTTTAAACTGGCTTTGAAGGAGTGTAAGCATGTTTTTGAAGGTTTGGGTTCTTTGGTTGATGTTGCTGGTGGTAGAGGTGGTGTTACAAAATTAATTAGGGAGGCTTTCCCACATGTTAAATGTACTGTTTTCGACCAACCACAAGTTGTTGCTAATTTGACTGGTGACGAAAATTTGAACTTCGTTGGTGGTGACATGTTTAAATCTGTTCCACCAGCTGATGCTGTTTTGTTGAAATGGGTTTTACACGATTGGAACGATGAATTATCCTTGAAAATCCTGAAGAACTGCAAGGAAGCTATTTCTGGTAGAGGTAAAGAAGGTAAAGTCATTATCATCGACATCTCTATCGACGAAACTTCTGATGATAGAGAATTGACTGAGTTGAAGTTGGATTACGATTTGGTTATGCTGACTATGTTCAACGGTAAAGAAAGAGAAAAGAAGGAGTGGGAAAAGTTAATCTACGATGCTGGTTTTTCCTCTTACAAAATCACACCAATCTGCGGTTTTAAGTCTTTAATCGAAGTCTTCCCATAA* |
| *AtGT* | *TTAAACTTTTTCTTTCTGCAGTTTAACCAGGGTCAGTTCTTCCATCAGACGATCAACTTCCAGATCAGAAGAGCCACCTTCTTTAACTGCGTTTTTCGCCATTTCAGCCAGTTCTTTCGCGCGTTTACGACGTTCTTCACCAACCATAACTTCACGAACCGCGCCTTCCACTTTTTCACGAGAAATGAAATCGCCAACAACCTGCATCATCTTTTTAACGCCAACGCTAACACCGGTTTTCAGAACCTGGGTAACCAGTTTTTCGTTGTAGAACTGTTCCGCACCAACCGGCCAGGTTACCATCGGCAGGCCCGCGGCAACACCTTCCAGCAGGCTGTTCCAGCCGCAGTGGGTCAGGAAACCGCCGATAGCTTTGTGTTCCAGAATCAGAACCTGCGGCGCCCAGCCACGGATGATCAGGCCTTTGCCTTTGGTTTTTTCTTCGAAGCCTTCCGGCAGCCAATCTTCTTTTTCAACCTGAGAACCTTTGCGGTTAACCACCCAAACGAAGTCATGGCCAGACATATCCAGGCCGGCTGCGATTTCGATCAGCTGTTCGTTTTTGAAAGAGGACATGGTGCCGAAAGCCATGTAGATCACGCTGTCGCATTTTTTGGAATCCAGCCATTTCAGACATTCGTGTTCATCGATGGACGCTTTTTTGCCACGTTCCGCTTTTTCTTCAAATTTGCGGTTACCCAGGCTCAGCGGACCGATGTGCCATGCACGTTTCGCAACGAAGCTTTTGAAATAGTCGCTATACGCCTGTTCCAGTTCGTAGAAAGAGTTAACCAGAACACCGAAGCTGTCACGTTCAGAATCACGGATCGCTTTCATGAAACGACCCATCACGCTTTCTTCTTCGGTTTCCATCACCTGTTCTTCAGTAATCAGGATGTCACCCGGCAGATCCGGGATCACGAACGGTTCAGAGCTGGTCGCTACGTTTTTCGGCAGACGAATGCAGTGGGACGCGCACAGGCTGAAGTAACCGGTGCCGTGGAACACCAGACGCGGCACACCGAATTTTTCCGCAACTTTGGTAGACCACGGGAAGAACATGTTACCAACCAGGCAATCCGGGCGCATGGTAACCAGCAGTTCTTCCAGCGGTTCTTCGAAGTATTTCATCGCCAGCAGAAATTTCTGGGACAGATCACCTACGTTCAGATCCGGGGTGCTGAAAATGAAATCGGTGTTTTCGCAACCATCCGGCAGACCCAGTTCGGTGCACGGGAAGTTCAGGATCTGAATGGTGATATCTTCCAGGCCCGGATTATCCTGGTTGAAGCTTTTGATCGGTTTTTCAAAGAACAGTTTAGCGTTCAGCGGGGTGGTCAGAATGGTGCTTTTCGCACCTTTGGTCGCGAACAGTTTTGCCATATCCAGGGTCGGGATCATGTGGCCGTGCGCCATGAACGGGAACAGCAGGAAGTGCAGTTTGCTAACTTCAACCGGGGTGCCCAT* |
| *Cm1,2-RhaT* | *ATGGATACTAAGCACCAAGATAAGCCATCTATTTTGATGTTGCCATGGTTAGCTCATGGTCATATTGCTCCACATTTGGAATTAGCTAAGAAGTTGTCTCAAAAGAATTTCCACATCTACTTTTGTTCTACTCCAAACAATTTGCAATCATTCGGTAGAAACGTTGAAAAGAATTTCTCTTCCTCTATTCAATTAATTGAATTGCAATTGCCAAACACATTCCCAGAATTGCCTAGTCAAAATCAAACTACTAAGAACTTGCCTCCTCACTTGATCTACACTTTGGTTGGTGCTTTTGAAGATGCTAAACCAGCTTTTTGTAATATTTTAGAAACTTTGAAACCAACTTTGGTTATGTACGATTTGTTCCAACCATGGGCCGCTGAAGCTGCCTATCAATATGATATTGCTGCTATCTTGTTCTTGCCATTGTCCGCTGTTGCTTGTTCCTTTCTATTGCATAACATTGTTAATCCATCTTTAAAGTATCCATTCTTTGAATCTGATTACCAAGACAGAGAATCAAAGAACATTAATTACTTTTTACATTTGACTGCTAATGGTACTTTGAACAAGGATAGATTTTTGAAGGCTTTCGAATTGTCTTGTAAATTTGTTTTCATTAAGACCTCTAGAGAAATTGAATCTAAGTATTTGGATTATTTTCCATCCTTGATGGGTAATGAAATTATTCCAGTTGGTCCATTGATCCAAGAACCAACCTTCAAGGAAGATGATACTAAAATTATGGACTGGTTATCTCAAAAGGAACCACGTTCAGTCGTCTACGCATCTTTCGGTTCTGAATACTTCCCATCCAAGGATGAAATTCATGAAATTGCCTCTGGTTTGTTGTTATCCGAAGTGAACTTTATTTGGGCTTTTCGTTTACATCCAGATGAAAAGATGACTATCGAAGAAGCTTTGCCTCAAGGTTTCGCTGAAGAAATTGAAAGAAATAATAAGGGTATGATTGTGCAAGGTTGGGTTCCACAAGCTAAGATTTTGAGACATGGTTCTATCGGTGGTTTCTTGAGTCATTGTGGTTGGGGTTCTGTTGTTGAAGGTATGGTTTTCGGTGTTCCAATTATTGGTGTTCCAATGGCATATGAACAACCATCTAACGCTAAGGTTGTTGTTGATAACGGTATGGGTATGGTTGTTCCAAGAGATAAAATTAACCAAAGATTAGGTGGTGAAGAAGTCGCTCGTGTTATTAAGCATGTTGTTTTACAAGAAGAAGCTAAACAAATTAGAAGAAAGGCTAATGAAATTTCCGAATCCATGAAGAAGATCGGCGATGCTGAAATGTCGGTTGTTGTTGAAAAATTGTTGCAATTAGTTAAGAAGTCTGAATAA* |
| *OlRHM* | *ATGGCTTCTCATACCCCAAAGAACATTTTGATTACCGGTGCCGCAGGTTTCATTGCTTCTCATGTTGCTAATAGATTGGTTAGAAAGTATCCACAATACAAAATTGTTGTTTTGGATAAATTAGATTACTGTTCTAACTTGAAGAACTTGAGACCTTCACAATTGTCCCCAAACTTTAAGTTCGTTAAAGGTGACATCGCTTCAGCCGACTTAGTTAATTATTTGTTGATTACTGAATCTATTGACACTATTATGCATTTCGCTGCTCAAACTCACGTTGACAACAGTTTCGGTAATTCTTTTGAATTCACAAAAAATAATATCTACGGTACTCACGTTTTGTTGGAAGCTTGTAAAGTTACTGGTCAAATTAAGCGTTTCATCCATGTTTCTACTGATGAAGTCTACGGTGAAACCGATGAGGACGCCGTAGTCGGTAACCACGAAGCTTCCCAATTGTTACCAACTAACCCATACTCTGCTACTAAGGCTGGTGCTGAAATGTTAGTTATGGCTTACGGTAGATCTTATGGTTTGCCAGTTATTACTACCAGAGGTAATAATGTTTATGGTCCAAACCAATTTCCTGAAAAGTTGATTCCAAAATTCATTTTATTAGCTATGAGAGGTAAATCTTTGCCAATTCATGGTGACGGTTCTAACGTCCGTTCCTACTTATATTGTGAAGATGTTGCTGAAGCTTTCGAATTAATTTTACATAAAGGTGAAGTTGGTCATGTTTATAACATTGGTACCAAAAAAGAAAGAAGAGTCATTGATGTAGCTAAAGACGTTTGTAAGTTATTCTCTTTGGATGCTGATTCCGTTATCAAATTCGTTGAAAATAGACCTTTCAATGATCAAAGATATTTCTTGGATGACCAAAAATTGACTAACTTGGGTTGGTCTGAAAGAACCACTTGGGAAGAAGGTTTGAGAAAGACTATGGAATGGTATACTTCTAATCCAGAATGGTGGGGAGATGTTTCTGGTGCCTTATTGCCTCACCCTCGTATGTTAATGATGCCTGGTATTGAAAAACAATTTGATGGCCCAGCTGATATTAACGGCACTTTGTCTGAATTAATGAAAAAGCCAACACAAACTGAAAAAGAAGTTGAAGCTTCTAAGAGAACCGCTAACTCACCACAAAAGCCAATGTTGAAATTTTTGATCTACGGTAGAACTGGTTGGATTGGTGGCTTACTTGGTCAAATTTGTGAAAAGCAAGGTATTCCATACGAATATGGTAAAGGTAGATTACAAGAAAGATCTCAATTAGTTAGTGATATTCAATCTGTTAAGCCAACTCATGTTTTTAACGCAGCAGGTGTGACTGGTAGACCAAATGTCGATTGGTGTGAATCCCACAAGCCAGAAACTATCAGAACTAATGTTGTTGGTACTTTGACATTAGCTGATGTCTGTAGAGAACACGGTTTGTTAATGATGAATTACGCTACTGGTTGTATATTCGAATACGACGCTCAACATCCAGAAGGTTCTGGTGTTGGTTTCAAAGAAGAAGATACACCAAATTTCGCTGGTTCTTTCTACTCCAAAACTAAGGCAATGGTCGAAGAATTGCTAAAGGACTATGATAACGTTTGTACTTTGAGAGTTAGAATGCCAATCTCCTCTGATTTGTCTAACCCTAGAAACTTTATTACTAAGATTTCTAGATACAACAAAGTTGTTAACATTCCAAACTCTATGACTGTTTTGGATGAATTGTTACCAATTTCAGTCGAAATGGCTAAGAGAAACTTGAGAGGTATCTGGAACTTCACTAATCCAGGTGTTGTTTCCCATAATGAAATCTTGGAAATGTATAAGAAGTACATGGATCCAGGTTTTAAGTGGTGTAATTTCACTTTGGAAGAACAAGCTAAGGTTATTGTTGCTGCTAGAAGTAATAACGAAATGGATGCCTCAAAGTTGAAAAAAGAATTCCCTGAATTGTTATCTATTAAGGACTCCTTGATTAAGTATGTTTTTGAACCAAATAAGAAGGTTTAA* |
| *vHb* | *ATGTTGGACCAGCAGACTATCAACATCATTAAGGCCACTGTTCCAGTTTTGAAGGAGCACGGTGTCACTATCACTACTACTTTCTACAAGAACTTGTTCGCTAAGCACCCAGAAGTTAGACCATTGTTCGATATGGGTAGACAAGAATCTTTGGAGCAACCAAAGGCTTTGGCTATGACTGTTTTGGCTGCTGCTCAAAACATCGAGAATTTGCCAGCTATCTTGCCTGCTGTTAAGAAAATTGCTGTTAAGCATTGTCAAGCTGGTGTTGCTGCTGCTCACTACCCAATTGTTGGTCAAGAATTGTTGGGAGCTATTAAGGAGGTTTTGGGTGATGCTGCTACTGATGACATTTTGGACGCTTGGGGTAAGGCTTATGGTGTTATCGCAGATGTTTTCATCCAAGTTGAAGCAGATTTGTACGCCCAGGCTGTTGAGTAG* |

**Table S3. Plasmids used in this study.**

| **Strain** | **Relevant genotype** | **Origin** |
| --- | --- | --- |
| pY26 | shuttle vector plasmid, Amp^R^, Ura 3 | This lab |
| pRS424 | shuttle vector plasmid, Amp^R^, Trp 1 | This lab |
| pRS426 | shuttle vector plasmid, Amp^R^, Ura 3 | This lab |
| P01 | pRS424-P_PGK1_-*MpOMT*-T_TPS1_ | This study |
| P02 | pRS424-P_PGK1_-*CrOMT6*-T_TPS1_ | This study |
| P03 | pRS424-P_PGK1_-*spnK*-T_TPS1_ | This study |
| P04 | pRS424-P_PGK1_-*SOMT2*-T_TPS1_ | This study |
| P05 | pRS424-P_PGK1_-*GeHI4'OMT*-T_TPS1_ | This study |
| P06 | pY26-P_ADH2_-*AtGT*-T_ADH1_ | This study |
| P07 | pY26-P_HXT7_-*AtGT*-T_ADH1_ | This study |
| P08 | pY26-P_ADH6_-*AtGT*-T_ADH1_ | This study |
| PCas1 | Cas9 gRNA-X3 | This study |
| PCas2 | Cas9 gRNA-XI3 | This study |
| PCas3 | Cas9 gRNA-XII5 | This study |
| PCas4 | Cas9 gRNA-XII1 | This study |
| PCas5 | Cas9 gRNA-ARO10 | This study |
| PCas6 | Cas9 gRNA-ARO9 | This study |
| PCas7 | Cas9 gRNA-PDC5 | This study |
| PCas8 | Cas9 gRNA-X4 | This study |
| PCas9 | Cas9 gRNA-DPP1 | This study |
| PCas10 | Cas9 gRNA-X2 | This study |
| PCas11 | Cas9 gRNA-EXG1 | This study |
| PCas12 | Cas9 gRNA-XII2 | This study |
| PCas13 | Cas9 gRNA-HO | This study |
| PCas14 | Cas9 gRNA-GAL1 | This study |
| PCas15 | Cas9 gRNA-XII3 | This study |
| PCas16 | Cas9 gRNA-XII4 | This study |
| PCas17 | Cas9 gRNA-EGH1 | This study |
| PCas18 | Cas9 gRNA-YPRCδ15C | This study |

**Table S4. gRNAs used in this study.**

| **Site** | **gRNA sequence** |
| --- | --- |
| *X2* | TCACTGAAAAAAGAAACGAG |
| *X3* | GACACATTAGTCTCGTATGT |
| *X4* | CGCCATTCAAGAGCAGCAAC |
| *XI3* | GTAGAAATCAGACGCACGCT |
| *XII1* | GGTATGTGCAGTTGATTCAC |
| *XII2* | TGAAACTCTAATCCTACTAT |
| *XII3* | CTTTATGCATAGAGCTAATT |
| *XII4* | CATGGTTTCAGAAAGCAGAG |
| *XII5* | TTGTCACAGTGTCACATCAG |
| *LPP1* | GAGATATCCCTGGTACCTAG |
| *HO* | TATGGAAGATACAAATTCAG |
| *GAL1* | TTAGAAAAGAGTGAGCAACA |
| *DPP1* | CGATTCAGATGTCACCCTGG |
| *PDC5* | TGCTTCATTGGAGTGTTAGC |
| *ARO9* | TAGCTTGTGAGGTACCACTG |
| *ARO10* | ATGGCCCTTCCTGCCGCCCT |
| *EXG1* | AACCGATAGGAATTCTGACA |
| *EGH1* | CACTTCGAATGACTTACCAG |
| YPRCδ15C | AATCCGAACAACAGAGCATA |

**Table S5. *Saccharomyces cerevisiae* strains involved in this study.**

| **Strain** | **Relevant genotype** | **Origin** |
| --- | --- | --- |
| CEN.PK2-1D | *MATα*; *ura3-52*; *trp1-289*; *leu2-3*,*112*; *his3Δ1*; *MAL2-8C*; *SUC2* | This lab |
| C800 | CEN.PK2-1D, *gal80::G418* | This lab |
| SQ05 | C800, *XI2*::P_GAL7_-*FjTAL*-T_DIT1_; *XI5*::T_CYC1_-*AtC4H*-P_GAL1,10_-*AtPAL2*-T_CIT2_-T_SDH1_-*AtATR2*-P_TDH3_-P_SED1_-*CYB5*-T_PDB1_ | This lab |
| XJ03 | SQ05, *X3*::T_ADH1_-*SjCHS1*-P_GAL10_-P_FBA1_-*MsCHI*-T_ADH2_-P_SED1_-*Pc4CL*-T_CYC1_ | This study |
| XJ04 | XJ03, *XI3*::T_ADH1_-*SjCHS1*-P_GAL10_-P_FBA1_-*MsCHI*-T_ADH2_-P_SED1_-*Pc4CL*-T_CYC1_ | This study |
| XJ06 | XJ04, *XII5*::T_ADH1_-*SjCHS1*-P_GAL10_-P_FBA1_-*MsCHI*-T_ADH2_-P_SED1_-*Pc4CL*-T_CYC1_ | This study |
| XJ07 | XJ06, *XII1*::T_ADH1_-*SjCHS1*-P_GAL10_-P_FBA1_-*MsCHI*-T_ADH2_-P_SED1_-*Pc4CL*-T_CYC1_ | This study |
| XJ08 | XJ07, *ARO10*::T_ADH1_-*SjCHS1*-P_GAL10_-P_FBA1_-*MsCHI*-T_ADH2_-P_SED1_-*Pc4CL*-T_CYC1_ | This study |
| XJ09 | XJ07, *ΔARO9* | This study |
| XJ10 | XJ07, *ARO9*:: P_ENO2_-*ARO4^K229L^*-T_IDP1_ | This study |
| XJ11 | XJ07, *ARO9*:: P_FBA1_-*ARO7^G141S^*-T_CYC1_ | This study |
| XJ12 | XJ07, *ARO9*:: P_ENO2_-*ARO4^K229L^*-T_IDP1_-P_FBA1_-*ARO7^G141S^*-T_CYC1_ | This study |
| XJ13 | XJ12, *ΔPDC5* | This study |
| XJ14 | XJ12, *ΔARO10* | This study |
| XJ15 | XJ12, *ΔPDC5*; *ΔARO10* | This study |
| XJ12-1 | XJ12, *X4*::P_TEF1_-*ALD6*-T_TPS1_ | This study |
| XJ12-2 | XJ12, *X4*::P_PGK1_-*ACS1*-T_PGK1_ | This study |
| XJ12-3 | XJ12, *X4*::P_PGK1_-*ACS2*-T_PGK1_ | This study |
| XJ12-4 | XJ12, *X4*:: P_TEF1_-*ALD6*-T_TPS1_-P_PGK1_-*ACS1*-T_PGK1_ | This study |
| XJ12-5 | XJ12, *X4*:: P_TEF1_-*ALD6*-T_TPS1_-P_PGK1_-*ACS2*-T_PGK1_ | This study |
| XJ12-6 | XJ12, *X4*:: T_TDH3_-*ALD6*-P_ADH2_-P_SSA1_-*SeACS^L641P^*-T_TAT1_ | This study |
| XJ12-7 | XJ12, *DPP1*:: P_TPI1_-*YlACL1-*T_TTYS1_ | This study |
| XJ12-8 | XJ12, *DPP1*:: P_PGK1_-*YlACL2-*T_PGK1_ | This study |
| XJ12-9 | XJ12, *DPP1*:: P_TPI1_-*AnACLa-*T_TTYS1_ | This study |
| XJ12-10 | XJ12, *DPP1*:: P_PGK1_-*AnACLb-*T_PGK1_ | This study |
| XJ12-11 | XJ12, *DPP1*:: P_TPI1_-*MmACL-*T_TTYS1_ | This study |
| XJ16 | XJ12-10, *X2*:: P_FBA1_-*ACC1^S659A, S1157A^-*T_TJID1_ | This study |
| XJ17-1 | XJ16, *X4*::P_PGK1_-*YHM2*-T_PGI1_ | This study |
| XJ17-2 | XJ16, *X4*::P_TEF1_-*PYC1*-T_TPS11_ | This study |
| XJ17-3 | XJ16, *X4*:: P_TPI1_-*IDP2*-T_TTYS1_ | This study |
| XJ17-4 | XJ16, *X4*:: P_TEF1_-*PYC1*-T_TPS11_-P_PGK1_-*YHM2*-T_PGI1_ | This study |
| XJ17-5 | XJ16, *X4*:: P_TPI1_-*IDP2*-T_TTYS1_-P_PGK1_-*YHM2*-T_PGI1_ | This study |
| XJ17-6 | XJ16, *X4*:: P_TEF1_-*PYC1*-T_TPS11_-P_TPI1_-*IDP2*-T_TTYS1_-P_PGK1_-*YHM2*-T_PGI1_ | This study |
| XJ18-1 | XJ17-6, pRS424-P_PGK1_-*MpOMT*-T_TPS1_ | This study |
| XJ18-2 | XJ17-6, pRS424-P_PGK1_-*CrOMT6*-T_TPS1_ | This study |
| XJ18-3 | XJ17-6, pRS424-P_PGK1_-*spnK*-T_TPS1_ | This study |
| XJ18-4 | XJ17-6, pRS424-P_PGK1_-*SOMT2*-T_TPS1_ | This study |
| XJ18-5 | XJ17-6, pRS424-P_PGK1_-*GeHI4'OMT*-T_TPS1_ | This study |
| XJ19-1 | XJ17-6, pRS424-P_PGK1_-*MpOMT*-T_TPS1_; pY26-P_ADH2_-*AtGT*-T_ADH1_ | This study |
| XJ19-2 | XJ17-6, pRS424-P_PGK1_-*MpOMT*-T_TPS1_; pY26-P_HXT7_-*AtGT*-T_ADH1_ | This study |
| XJ19-3 | XJ17-6, pRS424-P_PGK1_-*MpOMT*-T_TPS1_; pY26-P_ADH6_-*AtGT*-T_ADH1_ | This study |
| XJ20 | XJ17-6, *ΔEXG1* | This study |
| XJ21 | XJ17-6, *ΔEXG1*; pRS424-P_PGK1_-*MpOMT*-T_TPS1_; pY26-P_ADH6_-*AtGT*-T_ADH1_ | This study |
| XJ22 | XJ17-6, *ΔEXG1*; *XII2*:: P_PGK1_-*MpOMT*-T_TPS11_-P_ADH6_-*AtGT*-T_ADH1_ | This study |
| XJ23 | XJ22, *EXG1*:: P_SHM2_-*Cm1,2RhaT*-T_SDH1_; *LPP1*:: P_TDH1_-*OIRHM*-T_FBA1_ | This study |
| XJ24 | XJ23, *Ty4*:: P_PGK1_-*MpOMT*-T_TPS11_ | This study |
| XJ25-1 | XJ24, *HO*::P_TEF1_-*SAM1*-T_TPS1_-P_PGK1_-*MET6*-T_CYC1_ | This study |
| XJ25-2 | XJ24, *HO*::P_TEF1_-*SAM2*-T_TPS1_-P_PGK1_-*MET6*-T_CYC1_ | This study |
| XJ26-4 | XJ25-2, *GAL1*::P_CDC19_-*ADO1*-T_DIT1_ | This study |
| XJ26-5 | XJ25-2, *XII3*::P_GAL1,10_-*SAH1*-T_PGI1_ | This study |
| XJ26-6 | XJ25-2, *XII3*:: T_PGI1_-*ADO1*-P_GAL1,10_-*SAH1*-T_PGI1_ | This study |
| XJ27-2 | XJ26-5, *XII4*:: T_FBA1_-*STR2*-P_GAL1,10_-*MET2*-T_TPS1_ | This study |
| XJ28-2 | XJ26-5, *EGH1*:: T_TPI1_-*vHB*-T_PGI1_ | This study |
| XJ29-1 | XJ28-2, *YPRCδ15C*::P_CYC1_-*PGM2*-T_ter22_-*UGP1*-P_ARO7_ | This study |
| XJ29-2 | XJ28-2, *YPRCδ15C*::P_CYC1_-*PGM2*-T_ter22_-*UGP1*-P_INO1_ | This study |
| XJ29-3 | XJ28-2, *YPRCδ15C*::P_CYC1_-*PGM2*-T_ter22_-*UGP1*-P_ERG20_ | This study |
| XJ29-4 | XJ28-2, *YPRCδ15C*::P_SHM2_-*PGM2*-T_ter22_-*UGP1*-P_ARO7_ | This study |
| XJ29-5 | XJ28-2, *YPRCδ15C*::P_SHM2_-*PGM2*-T_ter22_-*UGP1*-P_INO1_ | This study |
| XJ29-6 | XJ28-2, *YPRCδ15C*::P_SHM2_-*PGM2*-T_ter22_-*UGP1*-P_ERG20_ | This study |
| XJ29-7 | XJ28-2, *YPRCδ15C*::P_SED1_-*PGM2*-T_ter22_-*UGP1*-P_ARO7_ | This study |
| XJ29-8 | XJ28-2, *YPRCδ15C*::P_SED1_-*PGM2*-T_ter22_-*UGP1*-P_INO1_ | This study |
| XJ29-9 | XJ28-2, *YPRCδ15C*::P_SED1_-*PGM2*-T_ter22_-*UGP1*-P_ERG20_ | This study |
